# Calculation of absolute molecular entropies and heat capacities made simple†

**DOI:** 10.1039/d1sc00621e

**Published:** 2021-03-25

**Authors:** Philipp Pracht, Stefan Grimme

**Affiliations:** Mulliken Center for Theoretical Chemistry, Institute for Physical and Theoretical Chemistry, University of Bonn Beringstr. 4 53115 Bonn Germany grimme@thch.uni-bonn.de +49-228-73-2351

## Abstract

We propose a fully-automated composite scheme for the accurate and numerically stable calculation of molecular entropies by efficiently combining density-functional theory (DFT), semi-empirical methods (SQM), and force-field (FF) approximations. The scheme is systematically expandable and can be integrated seamlessly with continuum-solvation models. Anharmonic effects are included through the modified rigid-rotor-harmonic-oscillator (msRRHO) approximation and the Gibbs–Shannon formula for extensive conformer ensembles (CEs), which are generated by a metadynamics search algorithm and are extrapolated to completeness. For the first time, variations of the ro-vibrational entropy over the CE are consistently accounted-for through a Boltzmann-population average. Extensive tests of the protocol with the two standard DFT approaches B97-3c and B3LYP-D3 reveal an unprecedented accuracy with mean deviations <1 cal mol^−1^ K^−1^ (about <1–2%) for the total gas phase molecular entropy of medium-sized molecules. Even for the hardship case of extremely flexible linear alkanes (C_14_H_30_–C_16_H_34_), errors are only about 3 cal mol^−1^ K^−1^. Comprehensive tests indicate a relatively strong variation of the conformational entropy on the underlying level of theory for typical drug molecules, inferring the complex potential energy surfaces as the main source of error. Furthermore, we show some application examples for the calculation of free energy differences in typical chemical reactions.

## Abstract 2

A novel scheme for the automated calculation of the conformational entropy together with a modified thermostatistical treatment provides entropies with unprecedented accuracy even for large, complicated molecules.

## Graphical abstract

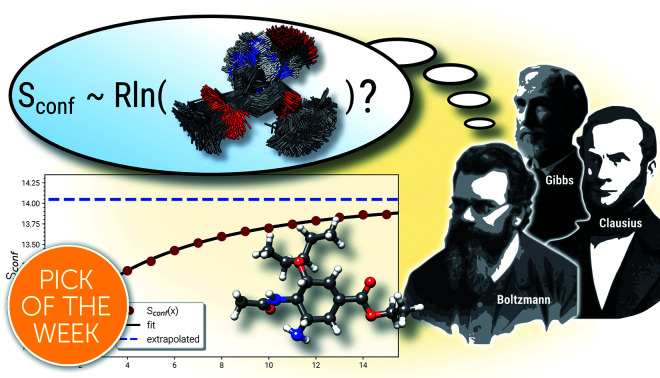

## 1 Introduction

A main goal of computational chemistry is to realistically model various chemical reactions and predict their products. While those reactions are usually carried out at room temperature in solution, quantum mechanical (QM) calculations are primarily conducted for isolated molecules at absolute temperature zero. In order to compare theory with experiment, additional corrections and computational steps are required. Calculations of thermodynamic properties at finite temperatures are essential and if we neglect here the issue of solvation, the basic problem is an efficient computation of the molecular entropy.^1,2^

As for most other thermodynamic properties, QM computations of the entropy are commonly based on frequency calculations in the harmonic oscillator (HO) approximation. This is then usually extended by the rigid-rotor model, giving rise to the rigid-rotor-harmonic-oscillator (RRHO) approach. A comparison of entropies calculated in this way to experimental values for small molecules reveals an insufficient accuracy already for relatively rigid molecules mainly due to anharmonicity effects.^3–6^ Because RRHO errors are often systematic, a common strategy is linear or multi-parametric scaling of the HO vibrational frequencies to mimic the effect of anharmonicity.^7–13^ However, even frequency scaling is unable to account for all of the missing contributions to the entropy.

Approaches that compute the absolute entropy can be roughly categorized into two major classes. The first go beyond the HO approximation and explicitly account for anharmonicities in the description mainly for low-frequency, torsional normal modes. For example, this can be done by construction of one-dimensional (1D) potential energy surfaces (PES) along the respective normal modes, as in the uncoupled normal mode approach of Sauer and coworkers.^14–16^ This scheme was later adapted by Head-Gordon *et al.*^6^ to include a separate treatment of vibrational and torsional modes (UM-VT). Advances have also been made for approaches that investigate coupled torsional motions.^17–19^ Another method that includes the torsional anharmonicity *via* 1D-PES and takes multiple structures into account is the MS-T approach (and its variants), developed by Truhlar and coworkers.^20–22^ Good results can be achieved with all of the above schemes, but in practice the construction of the PES and the relevant modes is technically involved, often only possible for relatively small molecules and unfeasible for routine computational chemistry workflows.

A stronger focus on multiple minima (molecular configurations/conformers) leads to the second class of approaches. Here, thermodynamic properties are approximated only by considering the *unique* minima on the PES, which in the molecular case are the different conformations. In the context of the mode following (MF) approaches discussed above, this can be understood because anharmonic torsional modes describe the transition between low-lying conformations.^23,24^ Although entropies and heat capacities are thermodynamic features encoded rather globally in the shape of the PES,^25,26^ conformations can be used to map the problem to well-defined points on the PES. More specifically, part of the absolute entropy is computed by an informational thermostatistic partition function (Gibbs–Shannon entropy^27,28^) that only depends on a given Boltzmann probability distribution of the conformers. This idea was pursued in the so-called “minima mining” approaches,^29–32^ where effects of anharmonicities are partially absorbed into the conformational entropy. As for the MF methods, a wide variety of different schemes exist,^33–36^ such as the so-called mutual information expansion (MIE),^37,38^ or the maximum information spanning tree (MIST)^39,40^ procedures. More recent developments were introduced by Suárez and coworkers.^41–43^ In their approach, the thermodynamic quantities are obtained from snapshots along an extended molecular dynamics (MD) trajectory, which are associated with unique molecular conformations. The vibrational contributions are averaged over all snapshots, while the configurational entropy is calculated *via* an MIE. This is doable at a force-field (FF) level, but will become cumbersome for medium sized drug-like molecules at higher theoretical levels. Note that essential parts of these schemes depend solely on structure based descriptors (dihedral angles). Other studies in the literature,^44^ employ some kind of flexibility measure to empirically derive molecular entropies and even more recently Hutchison *et al.* have used structural descriptors to develop a promising machine learned estimation of conformational entropy.^45^

In this study, we introduce an improved scheme that is developed from the minima mining approach and is designed to work in an almost “black box” fashion in combination with modified RRHO calculations. Herein, for the calculation of conformational entropies the recently developed GFN2-xTB^46,47^ tight-binding MO and GFN-FF^48^ force-field methods are employed to keep computational cost under control and improve the PES description in comparison to many standard FFs. Both methods are consistently available for all elements in the periodic table up to radon (*Z* = 86). Below, we will first start with a general overview of the partitioning of entropies and heat capacities, followed by a description of technical novelties and the automated procedure used for the conformational part. After discussing general observations with regard to entropy calculations, benchmark results for entropies and heat capacities are presented in comparison with experimental gas phase values. In the last section we apply our scheme to some biochemically relevant systems (drug molecules) and discuss a few prototypical chemical applications.

## 2 Theory

The absolute molecular entropy in the Born–Oppenheimer approximation consists of translational (trans), rotational (rot), and vibrational (vib, also termed internal) parts

1 *S* = *S*_trans_ + *S*_rot_ + *S*_vib_.

The most complicated vibrational contribution can be further decomposed according to

2 *S*_vib_ = *S*_HO_ + *S*_anharm_ + *S*_conf_,

where HO denotes the harmonic oscillator value, *S*_anharm_ its anharmonic correction and *S*_conf_ is the conformational entropy arising from the population of different conformational minima. This last term is relevant for many chemically important and often non-rigid molecules like alkanes or typical drugs. Its efficient computation is the main point of this work. The corresponding partitioning and formulas can be derived analogously for the heat capacity *C*_p_ for which only the finally used equation is reported below (see eqn (13)).

If *S*_anharm_ is neglected or as usually absorbed into a scaled *S*_HO_ term or partially accounted for by *S*_conf_ (see below), eqn (1) can be rewritten as

3 *S* = *S*_RRHO_ + *S*_conf_,

where *S*_RRHO_ refers to the usual rigid-rotor-harmonic-oscillator approximation for the rotational/translational and internal parts, respectively. In the following, in order to avoid terminology problems,^33^ we denote all parts of the entropy that are *not* included in *S*_RRHO_ (or *S*_msRRHO_, see below) of a given reference structure as *conformational* or *configurational* entropy and will use the terms interchangeably. The decomposition used above is physically motivated by the fact that some vibrational anharmonicity effects, at least for not too large distortions, maintain the equilibrium structure (bond stretching and many angle bendings), while many torsion motions lead to new (conformational) minima with low barriers. This partitioning of the entropy into vibrational and conformational parts was first introduced by Karplus *et al.*, and has since been used in many studies.^31,33,35,49–51^

A well-known problem of RRHO-based entropy calculations is that *S*_vib_ tends to infinity for vibrational frequencies approaching zero. In actual calculations for larger, flexible molecules, many low-frequency vibrational modes appear which are often better characterized by internal rotations of functional groups rather than by stretching or bending vibrations. They are in a typical range of 5–50 cm^−1^ and can spoil the computed entropy due to artificial numerical errors and their strong anharmonicity components. Correction schemes exist which explicitly treat such modes anharmonically in a coupled or uncoupled form.^6,22^ These methods require the costly computation of one-dimensional (1D) PES as well as definition of special internal coordinates. In our opinion, while such methods can be beneficial and accurate for small to medium sized and not too flexible molecules (≈20–30 atoms), they are not viable for a robust and rather general treatment for systems with hundreds of atoms.

In 2012, one of us proposed to modify the treatment of the low-frequency part of the vibrational spectrum by taking a so-called rotor-approximation and continuously interpolating between a rigid-rotor and vibrational description for each mode.^52^ Herein, the vibrational entropy of a harmonic oscillator with frequency *ν* at temperature *T* is given by4
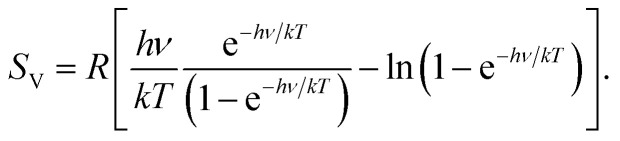


The rigid-rotor entropy for a free rotor is given by5
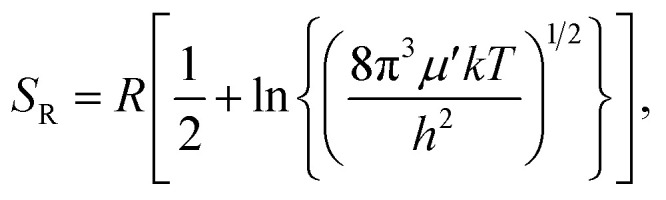
where *μ*′ describes the dependence on the average molecular moment of inertia *B*_av_ and the frequency of the normal mode6
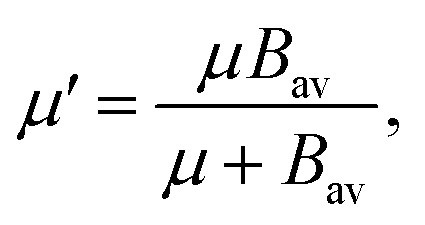
with 
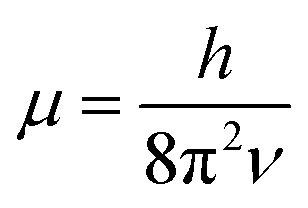
. In eqn (4)–(6), *h* is Planck's constant, *R* is the gas constant, and *k* is Boltzmann's constant. The final continuously interpolated *S*_mRRHO_ entropy (“m” for modified) is then given by a sum over all normal modes7
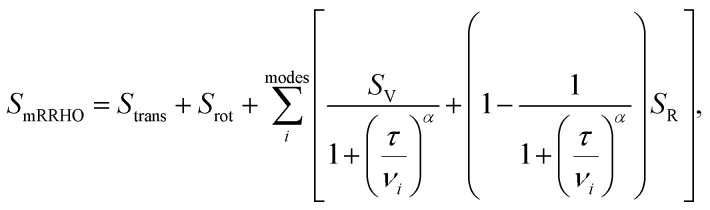
with *α* = 4 (introduced with the damping function in ref. 53). This does not involve any computational overhead compared to a standard HO calculation and merely requires the definition of a vibrational energy threshold *τ* below that the rotor entropy instead of the vibrational one is continuously taken. A related (but discontinuous) treatment has been proposed by Truhlar.^54^ A typical value used by us since years in standard thermochemical studies is *τ* = 50 cm^−1^. In this work, we consider *τ* for the first time as an adjustable parameter to account for part of the non-conformational anharmonicity effects. Furthermore, calculated harmonic frequencies are linearly scaled by a factor *ν*_scal_, as is common practice^7–9^ to account for deficiencies of the underlying method employed for the PES calculation and further anharmonicity effects mainly in the high-frequency part. The only two empirical parameters included are adjusted to reproduce experimental entropies for a benchmark set of mostly rigid molecules (see below). For better distinction this modified RRHO treatment is in the following denoted by *S*_msRRHO_ (“s” for scaled).

The major aim of this work was to find a robust approximation to *S*_conf_ which is already significant for medium flexible molecules (see Section 4.4). We build upon the original idea of Gilson and co-workers^29^ termed “minima mining” or “mixture of conformers” strategy, which has later been applied to organic molecule entropy calculations by DeTar^31^ and Guthrie.^32^ The basic formula reads8
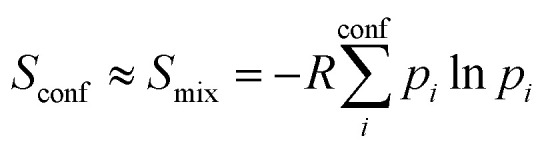
and approximates *S*_conf_ by the conformer mixing entropy *S*_mix_ summed over a conformer ensemble. The thermal populations *p* at absolute temperature *T* are given by9
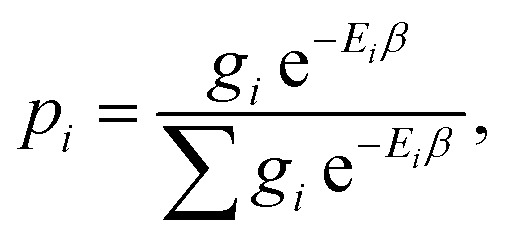
where 
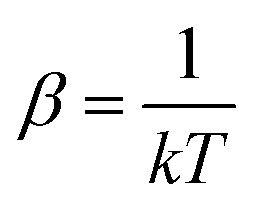
, *E*_*i*_ is the energy of the equilibrium structure of conformer *i*, and *g*_*i*_ is a general state degeneracy. The conformational entropy depends on the level of theory through the calculated populations entering the Gibbs–Shannon entropy formulation in eqn (8), which in turn depend directly on the equilibrium (free) energies. But also for other configurational entropy approaches, that are usually cited as being *purely informational*,^33,42^ there exists a bias towards the underlying method used for the generation of molecular structures, for example by MD simulations. This is especially problematic for very crude approximations of the conformational entropy, *e.g.*, based only on the number of conformers *N*_conf_ according to *S*_conf_ ≈ *R* ln(*N*_conf_). This approximation is used in some studies^32,55^ and is appealing due to its simplicity. However, while this formulation may be used for very simple molecules, it breaks down for more complex PES. Further discussion of this point is given in the ESI.†

The sum in eqn (8) is taken over all significantly populated, *distinguishable* structures representing a so-called generalized Boltzmann distribution.^28^ The problem of this procedure (also termed Gibbs–Shannon entropy based procedure) is that not only an almost complete conformer ensemble has to be found but additionally, it should be “pure”, *i.e.*, free of so-called rotamers. In this case for molecules with non-degenerate electronic ground states, all *g*_*i*_ are unity. Rotamers are structures indistinguishable by any nuclear spin-independent quantum mechanical observable. They arise from rotation around covalent chemical bonds (or other inversion-type processes) that interchange nuclei belonging to the same group of nuclides, as for example the interchange of protons at a methyl group by rotation.

In this work, we propose and implement for the first time an automatic algorithm that generates a theoretically proper ensemble of unique conformer structures required for the accurate computation of *S*_conf_. For the conformer search problem, we employ our recently described CREST program^56^ (abbreviated from Conformer-Rotamer Ensemble Sampling Tool), which is based on metadynamics simulations employing on-the-fly computed quantum mechanical tight-binding PES.^56,57^ We assume at this point that the conformer-rotamer ensembles (CRE) obtained from CREST are sufficiently complete and the energies *E*_*i*_ are accurate. If this is really the case for very flexible molecules (*e.g.* long alkanes) can be tested by comparison of computed and experimental entropies and heat capacities (see Sections 4.2 and 4.3). Note that our approach works with any (on-the-fly computed) PES and hence, at least in principle, the errors introduced by the underlying method for the PES and the other approximations to the entropy problem could be decomposed.

The CREST algorithms were originally developed to generate rotamer containing ensembles and the related nuclei-exchange information for the simulation of NMR spectra.^23^ Hence, it seems straightforward not only to identify rotamers, but to extend the algorithm to automatically compute the proper degeneracy number *g*_*i*_. However, as mentioned above, conformer ensembles (CE) must be free from the indistinguishable rotamers to be compatible with entropy calculations. Therefore, *g*_*i*_ are treated as unity in the usual case.

The only exception here are symmetrical molecules that can form “enantiomeric” (*i.e.*, in principle distinguishable) conformers through rotation of bonds. A typical case is the gauche conformer of *n*-butane. These geometrical enantiomers are degenerate and would be falsely classified as rotamers in our previous implementation. Effectively, this introduces a factor of 
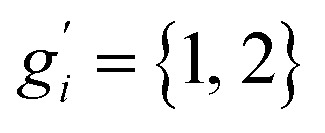
 instead of *g*_*i*_ in the degeneracy, depending on if the formation of a geometrical enantiomer is possible. Our new approach considers this problem for the first time in a correct and automated way. Inserting this into the standard entropy expression for degenerate states^58^ leads to10
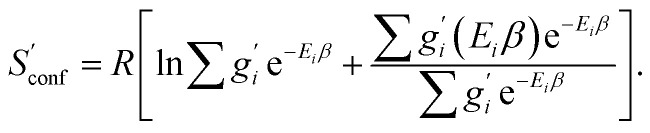


The correct *S*_msRRHO_ entropy is a population average over the CE, analogously to other physical observables. Unfortunately, the many costly DFT geometry optimizations and frequency calculations will quickly become the computational bottleneck for moderately sized systems. Therefore, as a further approximation, we compute *S*_msRRHO_ at the DFT level for the lowest conformer and add the respective ensemble contribution as a thermostatistical average over all populated conformers at a less computationally demanding, lower theoretical level. The arising *S̄*_msRRHO_ term is given by

11 *S̄*_msRRHO_ = (∑*p*_*i*_*S*_msRRHO,*i*_) − *S*_msRRHO,ref_,

where *S*_msRRHO,*i*_ is the absolute msRRHO entropy of the conformer calculated at the low force-field or SQM level to avoid very many (high level/DFT) HO calculations. *S*_msRRHO,*i*_ and the free energies (*G*_*i*_) are only explicitly calculated for the lowest ≥90% populated (based on initial total energies *E*_*i*_) conformers while for all others, the average is taken. The populations *p*_*i*_ refer to eqn (9) and are calculated using *G*_*i*_ from the corresponding msRRHO calculations. For convenience, we subtract the entropy of a reference structure *S*_msRRHO,ref_ in eqn (11) such that *S̄*_msRRHO_ can be added directly taken as a further correction to the *S*_mRRHO_ result taken from any standard quantum chemistry code. *S*_msRRHO,ref_ typically refers to the DFT reference structure, for which vibrational frequencies are calculated at the SQM or FF level. To avoid changes to the geometry and appearance of imaginary vibrational modes, we here additionally make use of a new procedure called Single Point Hessian (SPH),^59,60^ for which some details are given in the ESI.† Note that if *S̄*_msRRHO_ is calculated at the same level as *S*_msRRHO_, one would arrive at the correct population average because *S*_msRRHO_ and *S*_msRRHO,ref_ exactly cancel each other. The treatment would then be exact.

Thus, our final working equation for the molecular entropy is given by12
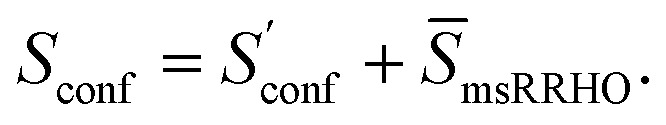


The corresponding formula for the heat capacity at constant pressure is13
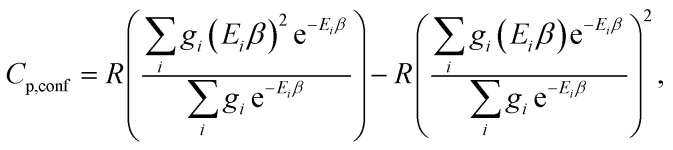
and the enthalpy is14
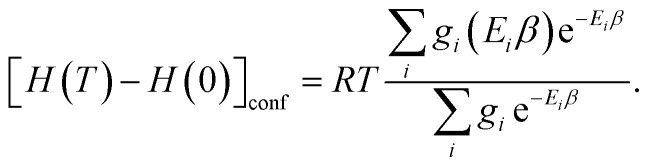


Note that *g*_*i*_ is used in *C*_p_ and *H*(*T*) − *H*(0) instead of 
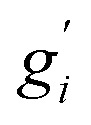
. In our opinion, basing *S*_conf_ (and related properties) directly on a given level of theory *via* the Gibbs–Shannon entropy of an ensemble (eqn (8) and (10)) provides a genuine understanding of the quantity in accordance with chemical intuition. Furthermore, it can be very well coupled to automated conformational search tools, which are anyway necessary for accurate computation of other physical observables.

## 3 Implementation and computational details

### 3.1 Extrapolation to ensemble completeness

For very flexible systems (*e.g.* long alkanes), the number of accessible conformers *Ω* is roughly proportional to *Ω* ≈ 3^*R*^, where *R* is the number of freely rotatable bonds (commonly associated with the number of sp^3^–sp^3^ carbon single bonds).^55^ In principle, all conformers, *i.e.*, the complete ensemble and the respective energies are required for the calculation of *S*_conf_ but even for only moderately sized systems this number is prohibitively huge.

Practically, the obtained ensemble quality depends mostly on the run time *t* of the (biased) molecular dynamics (MD) in CREST. Basically, it is the number of optimized snap-shot structures gathered over all runs and will converge to a complete CE with the length of the conformational search. On the other hand, the conformational entropy also exhibits predictable behavior with regard to increasing ensemble completeness. If the lowest energy conformer is known, adding higher-lying conformers to the ensemble can only increase the entropy. If many of the low-energy structures are already found, the entropy increase for additional states is smooth and it seems possible to extrapolate to completeness without explicit knowledge of all conformers. The pre-requisite for this is the generation of enough intermediate points, *i.e.*, consecutive conformational ensembles with systematically improved quality. A smooth and continuous convergence of the entropy to its maximum value can only be observed if conformers are added consistently from all regions of the PES (see Section 4.2 for examples).

In the implementation of the algorithm, information from incomplete CEs of consecutive iterations is used for an extrapolation of the entropy according to15
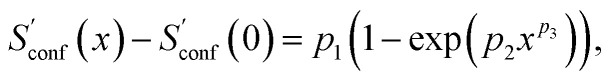
where *x* is the iteration number, and 
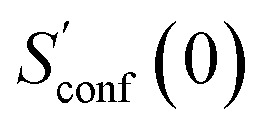
 refers to the result of the first initial conformer ensemble from the new CREST workflow (see Section 3.2). The parameters *p*_1_, *p*_2_ and *p*_3_ are fitted automatically to the available data points from each entropy sampling run employing the Levenberg–Marquadt^61,62^ algorithm. In summary the extrapolation can be seen as an unsupervised learning procedure used to correct for incompleteness.

### 3.2 Algorithmic and technical details

The conformational entropy calculation as described above is performed with the recently published CREST program.^56^ A special run type was implemented for this purpose, where the focus is set to an extensive sampling around the global and low-lying local minima. Ideally the calculation of *S*_conf_ should be conducted from the already known global minimum conformer, *e.g.*, obtained from another conformational search with default settings in CREST. The enantiomer degeneracy number *g*_*i*_ is obtained automatically as described in detail the ESI.† For the msRRHO part, any quantum chemical method or even force-fields can be applied. Here, we use the composite DFT method B97-3c^63^ and the well-known B3LYP-D3 functional^64–66^ in a standard def2-TZVP basis.^67^ Molecular symmetry numbers are automatically determined for each conformer entering *S̄*_msRRHO_ and should be also included in the DFT frequency evaluation.

The few simple steps required for the calculation of the absolute entropy are

(1) Run CREST in default mode on a starting structure to find the lowest conformer.

(2) Optimize the geometry of this conformer with DFT, compute the Hessian matrix from the DFT structure and use the HO vibrational frequencies to calculate *S*_msRRHO_.

(3) Run CREST in entropy mode on the lowest-energy conformer and employ the DFT reference structure for *S̄*_msRRHO_, resulting in *S*_conf_.

(4) Compute *S* = *S*_msRRHO_ + *S*_conf_.

Note that for large systems step two could in principle also be conducted at a low theory level (SQM or FF). However, because step three is usually the computational bottleneck, it is recommended to take *S*_msRRHO_ from a more accurate DFT treatment. In general, this partitioning allows systematic improvements of the scheme because the different contributions can in principle be calculated at any level of theory.

If no low-lying conformers (relative energy < 1–2 kcal mol^−1^ at ambient temperature) are found in the first step, the entropy run is not necessary and the plain *S*_msRRHO_ value can be taken. The default setup for the metadynamics bias potentials in the entropy mode and further technical settings were empirically determined on a few test cases similar to the optimization of the run parameters in a conventional conformer search run^57^ (see CREST documentation and source code^68^). Note that the MD runs are by default initiated with random numbers and hence the details of the obtained CE vary stochastically. For larger, very flexible molecules with a complicated PES this can amount to stochastic variations of 2–5% for *S*_conf_ (see also Section 4.4 for discussion).

**Fig. 1:**
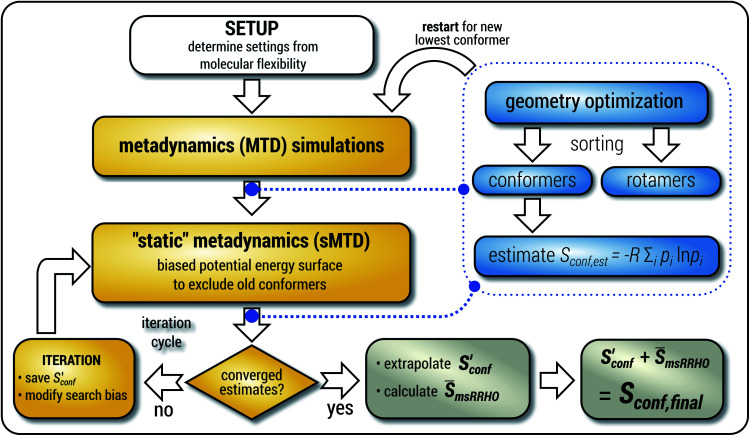
Schematic representation of the workflow used for the computation of *S*_conf_. See text for details.

The general workflow for the computation of *S*_conf_ in CREST is outlined in Fig. 1.

The procedure is designed to work fully automatic and to provide intermediate ensembles for entropy extrapolation as described above. For the input structure, the run time *t* of the biased MD is determined automatically from a covalent and non-covalent flexibility measure (see Section 4.4 and the ESI†). To create an initial structural ensemble, 24 metadynamics (MTD) simulations are conducted with several different bias parameters as in the default CREST runtype. The structural ensemble obtained from this step is later used as the reference to calculate 
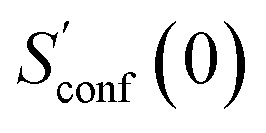
 (see eqn (15)). Structures are sorted according to their relative energy, structural Cartesian RMSD, and rotational constants to distinguish between unique conformers and degenerate rotamers, as described in ref. 56.

From the CEs two sets of structures are extracted *via* a combined principle component analysis (PCA)^69,70^ and k-means clustering^71,72^ approach, using dihedral angles as geometrical descriptors. The first set of structures, which always consists of 36 structures, is used as input for further metadynamic simulations. The other set consists of a number of structures that depends on the molecular flexibility and current ensemble size. This second ensemble is used to generate a global bias potential in the metadynamics simulations and, in contrast to the initial MTD simulation, is not updated with new bias structures. The idea here is to apply this new unchanged bias similar to a global potential used in classical umbrella sampling^73^ or basin-hopping algorithms^74,75^ to efficiently block entire energy basins of the PES and direct the conformational search to new minima. For better differentiation, this is referred to as static metadynamics simulation (sMTD). The ensemble obtained by sMTD is merged with the previous ensemble and a preliminary conformational entropy *S*_conf,est_ is determined. If no change (within a 0.5% threshold) in *S*_conf,est_ and the total number of unique conformers (within 2%) is observed, the final conformational entropy is calculated. Otherwise, a new iteration of 36 sMTDs is conducted using input structures and static bias structures determined from the updated ensemble. Furthermore, with each iteration the number of static bias structures is increased. This procedure is repeated until convergence is reached both with regards to *S*_conf,est_ and the number of unique conformers in the ensemble. For the final calculation of 
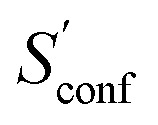
, an extrapolation as described in Section 3.1 is conducted. This new algorithm in CREST can also be used for normal conformer search with the keyword –v4. The default convergence thresholds were conservatively chosen to provide good reproducibility (see Section 4.4), but can manually be adjusted.

A problem may appear if the rather approximate PES used in CREST (here GFN2-xTB or GFN-FF) is substantially different from the DFT PES (here B97-3c or B3LYP-D3/def2-TZVP). This is indicated by different lowest-energy conformers and significant energetic re-ordering of the CREST ensemble obtained with the GFN methods after refining (re-optimizing) it with the respective DFT methods. In such cases, we suggest to use the *S*_msRRHO_ value obtained for the lowest DFT conformer and corresponding *S*_conf_ from the GFN ensemble. If the lowest GFN and DFT conformer structures agree qualitatively, this approximation seems to be reasonable according to our experience.

Ideally, the PES employed for the initial conformational search and the one used for automatic *S*_conf_ calculation should be the same. Here, we employ the GFN2-xTB tight-binding method^46^ and the recent general force-field GFN-FF^48^ and compare the results. The latter speeds-up the CREST calculations by a factor of 10–30 for typical cases with 50–100 atoms. The *S*_msRRHO_ value is always computed with B97-3c and a frequency scaling factor *ν*_scal_ of 0.97, or B3LYP-D3/def2-TZVP with a frequency scaling factor *ν*_scal_ of 0.98. Test calculations employing GFN2-xTB in this step yield somewhat less accurate results and, because the calculation of *S*_conf_ is the computational bottleneck, do not reduce the overall computational times significantly. In all frequency calculations, a *S*_msRRHO_ cut-off value of *τ* = 25 cm^−1^ was employed. *τ* and *ν*_scal_ (for the DFT methods) were adjusted to perform equally well in combination with both GFN-FF and GFN2-xTB. CREST is essentially a driver for the xtb program^76^ which is used for all GFN calculations. For the DFT calculations, TURBOMOLE 7.4 (ref. 77 and 78) is used throughout.

### 3.3 Benchmark sets

For the initial tests and determination of the empirical parameters *τ* (msRRHO cut-off) and *ν*_scal_ (DFT frequency scaling factor) we employ the benchmark set of Li, Bell and Head-Gordon (LBH).^6^ This LBH set consists of 39 organic molecules ranging from ethane (smallest) to *n*-octane (largest) and is shown in the ESI.† For cross-validation we extended this set by 23 similar, but mostly larger molecules ranging from cyclohexane (smallest) to *n*-dodecane (largest). This set is termed AS23 (absolute entropy) from now on and is described also in the ESI.† The corresponding experimental gas phase reference entropies and *C*_p_(*T*) values are taken from ref. 79 and 80. Studies are available in the literature presenting much larger collections of experimental reference data, *e.g.*, in ref. 55. However, these databases contain mostly small, rather rigid systems (*e.g.*, substituted aromatic compounds) which are not in the focus of our study. Nonetheless, the combined LBH and AS23 sets should sufficiently representative for benchmarking absolute entropies. To show possible limitations of our approach a set of maximally flexible linear alkanes (up to C_18_H_38_) is investigated separately.

For the heat capacities, we additionally test the temperature dependence in a typical range of 200–1500 K, while for entropies only the value at 298 K is considered. For this a subset of the LBH molecule set is used, as described in ref. 6. Note that the numerical values and errors for entropy and *C*_p_ are similar and thus, the conclusions for the temperature dependence of the latter should also apply for the entropy.

Furthermore, in Section 4.4 we present a case study for 25 pharmaceutical (clinical drug) molecules, denoted CD25. There are no experimental entropy values available for this set, but differences between the ensembles (*e.g.*, gas phase *versus* implicit solvation) and different PES employed to calculate the entropy can be studied theoretically. We suggest this set also as a challenging test for other approaches.

## 4 Results

### 4.1 General considerations

The absolute entropy is a complicated property which includes various terms of different magnitude that can be qualitatively interpreted.^29,33^ As an example the suggested partitioning of the absolute entropy for two molecules is shown in Table 1.

**Table 1:** Contributions to the total molecular entropy for *n*-decane and tamiflu. RRHO and msRRHO values correspond to the B97-3c level of theory, 
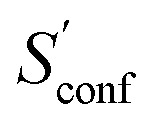
 and *S̄*_msRRHO_ were calculated at the GFN2-xTB level. Relative contributions are given in percent next to the respective contribution

		*S* (cal mol^−1^ K^−1^)	
		*n*-Decane	Tamiflu
RRHO		116.4	169.0
msRRHO		117.3 (89.9%)	173.4 (91.6%)
	vib.	47.2	95.4
	rot.	29.4	34.9
	trans.	40.8	43.1
Anharm. (msRRHO-RRHO)		0.9	4.4
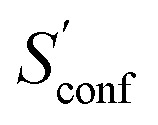		12.5 (9.6%)	13.7 (7.2%)
*S̄*_msRRHO_		0.7 (0.5%)	2.3 (1.2%)
Sum		130.5 (100.0%)	189.4 (100.0%)
Exptl.		130.4	—

The largest portion of the entropy results from the vibrational, rotational, and translational degrees of freedom (DOF), as commonly obtained by standard quantum mechanical frequency calculations employing the RRHO approximation. Contributions from translational and rotational DOF have the same order of magnitude (about 30–40 cal mol^−1^ K^−1^ in Table 1) for all chemical systems of about this size (mass). In contrast, vibrational contributions quickly exceed several hundred cal mol^−1^ K^−1^ for molecules >100 atoms. In the important drug-size regime, the vibrational entropy is clearly the largest contribution and hence its accuracy depends also on how good anharmonicities are described. As defined in Section 2, the effect of anharmonicities can be estimated from the difference between the entropy calculated by the new msRRHO and standard RRHO scheme (*i.e.*, without modifying *τ* and frequency scaling). Looking at the two example molecules, decane shows only a relatively small RRHO-msRRHO difference of 0.9 cal mol^−1^ K^−1^ while tamiflu exhibits a much higher anharmonic contribution of 4.4 cal mol^−1^ K^−1^. This is in line with chemical intuition, as one would expect many more anharmonic ro-vibrational modes for a complicated drug molecule like tamiflu than for a rather simple linear structure composed of only CH and CC bonds. In any case, the anharmonicity is non-negligible and must be accounted for by either *τ* and *ν*_scal_ or some more elaborate, explicit scheme. With increasing flexibility of the molecule the configurational contribution increases drastically and in fact, *S*_conf_ can be taken as a molecular flexibility measure (see Section 4.4).

For decane and tamiflu the conformational entropy 
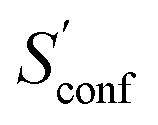
 accounts for 12.5 and 13.7 cal mol^−1^ K^−1^, respectively. Though decane (32 atoms) is smaller than the drug molecule tamiflu (50 atoms), their conformational entropy values are rather similar. The simple explanation for this is the higher flexibility of decane, which is typically indicated by a larger relative contribution of 
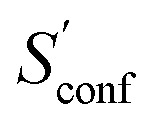
 to the absolute entropy for similar sized structures. In general 
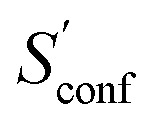
 will be close to zero for the most rigid molecules or molecules with only a few distinct conformers, but adds a significant portion (ten or more percent) to the absolute entropy for highly flexible molecules.

**Fig. 2:**
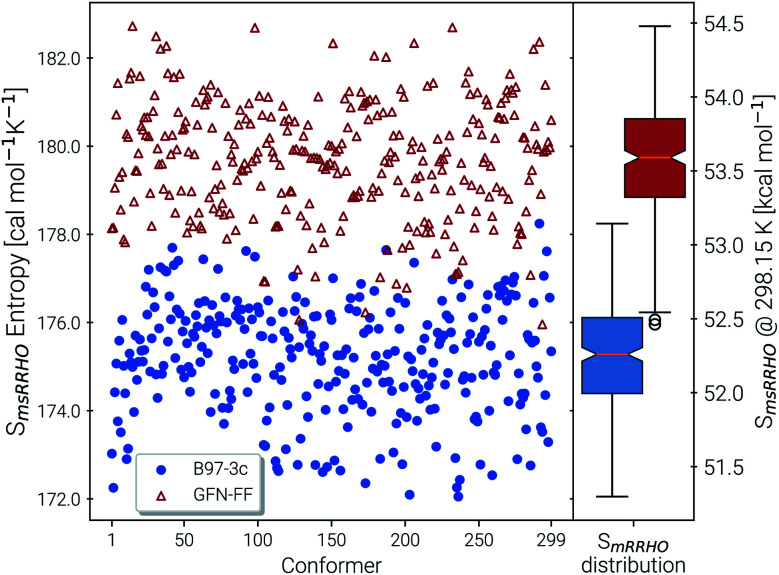
Spread of entropies calculated in the msRRHO approximation at the GFN-FF (red) and B97-3c (blue) level. On the right side box plots for the two methods are given for an easier visualization of the metric averages and shifts.

The last contribution to *S*_conf_ is the population average *S̄*_msRRHO_. This term may provide insight about the variation of *S*_msRRHO_ within the ensemble. It will be small if all contributing conformers have a similar ro-vibrational entropy as the reference structure (*e.g.* for decane with 0.7 cal mol^−1^ K^−1^), or yields a large contribution in the opposite case (tamiflu, 2.3 cal mol^−1^ K^−1^). For the latter, computed msRRHO entropies can vary by several entropy units for different conformations rather independently of the chosen *τ* or *ν*_scal_ values. An example is provided in Fig. 2, where *S*_msRRHO_ was calculated for 299 (random) conformers of tamiflu at two different theoretical levels (GFN-FF and B97-3c).

Here, entropies at the GFN-FF level are overestimated by 4 cal mol^−1^ K^−1^ on average compared to the more accurate B97-3c level. Both methods show a similar spread of the *S*_msRRHO_ values, which range approximately 6 cal mol^−1^ K^−1^ from lowest to highest value thus reconfirming the use of *S̄*_msRRHO_. Hence, the validity of an approximate *S̄*_msRRHO_ obtained at SQM or FF level depends on the performance for *relative* msRRHO entropies and may be used if a shifted (*cf.*eqn (11)) population average similar to the higher reference DFT level is expected.

**Fig. 3:**
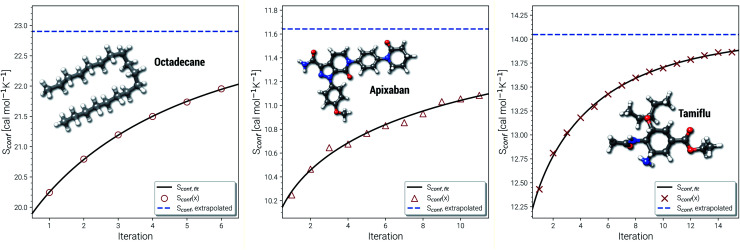
Examples for the extrapolation of conformational entropy at the GFN-FF level of theory. The iteration number *x* refers to the sMTD iteration cycle depicted in Fig. 1.

Another novelty of our approach is the extrapolation of 
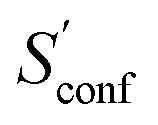
 to the ensemble completeness as discussed in Section 3.1. The corresponding procedure requires systematically and smoothly improving CE quality in each iteration. In practice, the required number of iterations is very molecule specific but convergence is typically achieved within 5–15 iterations (see Fig. 3 for some examples).

The entropy difference between the last iteration and the extrapolated value is often relatively small but very significant for very flexible systems with huge ensembles. For example the CE of *n*-octadecane contains over half a million conformers within 6 kcal mol^−1^ at the last iteration. In a more typical case the entropy gain due to the extrapolation is smaller than one entropy unit (1 cal mol^−1^ K^−1^). Apixaban and tamiflu depicted in Fig. 3 are such examples, but nonetheless exhibit different convergence behavior. For small molecules the extrapolation is mostly not necessary because the entire ensemble will be found during the initial sampling procedure. From another viewpoint, the extrapolation scheme might rather be seen as a technical supplement for reduction of stochastical noise between the iterations and consequently, an improved prediction the final *S*_conf_ value. Note, that 3 cal mol^−1^ K^−1^ ”entropy units” refer to the usual 1 kcal mol^−1^ chemical accuracy at room temperature. Thus, with an accuracy for *S* better than about 1–2 cal mol^−1^ K^−1^, the electronic energies of the molecules from DFT or wave function theory (WFT) become the accuracy bottleneck in typical thermochemical calculations.

### 4.2 Benchmarking absolute entropy

**Fig. 4:**
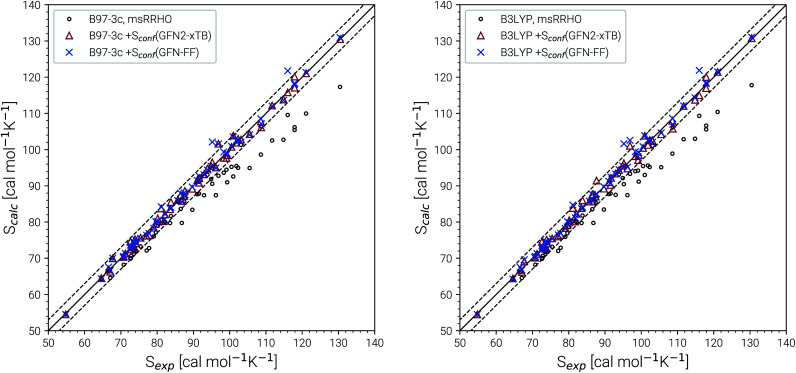
Parity plots for calculated and experimental entropies for all molecules of the LBH and AS23 set. The combinations of B97-3c and B3LYP-D3/def2-TZVP *S*_msRRHO_ values with GFN2-xTB and GFN-FF *S*_conf_ values, respectively are shown. For reference also the plain *S*_msRRHO_ entropies are plotted. The solid line corresponds to perfect correlation between theory and experiment. Error bars of 3 cal mol^−1^ K^−1^ are given as dashed lines and correspond to chemical accuracy at *T* = 298 K.

Recently, Head-Gordon *et al.* published the LBH set containing 39 organic molecules and their experimental gas-phase entropies, which provides an excellent reference for the evaluation of absolute entropies.^6^ For a more thorough evaluation the set was extended by the AS23 molecules. Entropy values for the two sets were calculated for four combinations of theory levels. These are *S*_msRRHO_ contributions obtained with either B97-3c or B3LYP-D3/def2-TZVP and the conformational entropies calculated at GFN-FF or GFN2-xTB level and with *τ* and *ν*_scal_ values as described above. Parity plots for the different levels of theory with reference to the experimental data are given in Fig. 4 and the corresponding statistical data are provided in Table 2.

**Table 2:** Mean deviation (MD), mean average deviation (MAD), root-mean-square deviation (RMSD), and standard deviation (SD) for absolute entropies obtained at different theoretical levels in comparison to experimental data. All values correspond to standard entropies at 298.15 K in cal mol^−1^ K^−1^. Three outliers have been removed for the final GFN-FF results (see text)

*S*_RRHO_	B97-3c		B3LYP-D3/TZ		UM-VT^a^
*S*_conf_	GFN-FF	GFN2-xTB	GFN-FF	GFN2-xTB	
**LBH set**					
MD	0.32	0.23	0.23	0.09	−0.52
MAD	0.59	0.65	0.60	0.65	0.86
RMSD	0.84	0.91	0.85	0.93	1.24
SD	0.79	0.89	0.83	0.93	1.14

**Full set**					
MD	0.21	0.15	0.24	0.07	—
MAD	0.73	0.83	0.73	0.92	—
RMSD	1.09	1.19	1.16	1.29	—
SD	1.08	1.19	1.15	1.30	—

a Values taken from ref. 6.

The excellent performance of our approach is obvious from both Table 2 and the parity plots (Fig. 4). To the best of our knowledge, the RMSD of 0.79 cal mol^−1^ K^−1^ calculated at the B97-3c + *S*_conf_(GFN-FF) level refers to the best performance of a theoretical method for this benchmark set ever reported in the literature. For comparison, the best performing method discussed in ref. 6 (UM-VT, a DFT based MF approach) has a RMSD of 1.24 cal mol^−1^ K^−1^. For the combined LBH + AS23 set the errors are slightly larger (RMSD of 1.1–1.3 cal mol^−1^ K^−1^). Yet, all of the four tested method combinations are well below the targeted chemical accuracy of 3 cal mol^−1^ K^−1^. A similar performance on a set of 128 experimental absolute entropies was reported by Guthrie^32^ using B3LYP/6-31G**, with an RMSD of 1.29 cal mol^−1^ K^−1^. Larger, flexible molecules in this set are identical with the ones in the LBH + AS23 set. However, Guthries benchmark set is mainly composed from rather rigid structures for which the *S*_RRHO_ entropy is already quite accurate.

For both B97-3c and B3LYP-D3, deviations between the calculated *S*_msRRHO_ (or *S*_RRHO_ values, data not shown) and the experimental value increase with the size and flexibility of the molecule. Only by including the conformational contributions it is possible to reach chemical accuracy. Overall, the different method combinations show fairly similar performance, although some trends can be recognized. A good performance of B3LYP-D3 is unsurprising as it is well known to be among the best performing DFT functionals for the calculation of vibrational properties^7,8^ and was basically constructed for this purpose.^64^ Although the (computationally cheaper) B97-3c method performs slightly better than B3LYP-D3/def2-TZVP, this is sensitive to the choice of *τ* and *ν*_scal_ and furthermore depends on the technical settings of the DFT calculations, like the choice of the grid or SCF convergence thresholds.^81^ Therefore, a clear preference for one out of the two tested methods is difficult to draw.

The same is true when comparing the two assessed methods for calculating *S*_conf_. *S*_conf_ strongly depends on the shape of the PES which can be rather different between a force field and a quantum chemical method. Since GFN2-xTB has the more physically reasonable PES of the two methods, usually a better performance should be expected. However, GFN-FF seemingly outperforms GFN2-xTB in combination with both B97-3c and B3LYP-D3 but this is mainly due to the removal of three strong outliers (3,3-dimethylpentane, 3,3-diethyl-2-methylpentane and perfluoroheptane) that were discarded from the GFN-FF error statistics. For all three molecules GFN-FF produces some artificially low-lying conformers resulting in an overestimation of the conformational entropy (7%, 5% and 3% respectively). Only one additional outlier, triethylamine (TEA), is observed for the combined LBH + AS23 set, but since it is present for all four method combinations, it may not be attributed to a wrong conformational energy landscape. The origin of the error for TEA (overestimation by approximately 5%) remains unknown, but it has not been removed from the statistics presented in Table 2. Without TEA the statistics would improve even further to low MADs and RMSDs of 0.77 and 1.04 cal mol^−1^ K^−1^ for B97-3c and 0.87 and 1.18 cal mol^−1^ K^−1^ for B3LYP-D3 in combination with *S*_conf_(GFN2-xTB), respectively. The best overall result for the LBH + AS23 set after removing all outliers is obtained with B97-3c + *S*_conf_(GFN-FF). Interestingly, our *S*_msRRHO_ + *S*_conf_ values tend to slightly overestimate compared to the experimental data, while the opposite holds for approaches that go beyond the harmonic approximation, such as UM-VT.^6^ This is indicated by the mean deviation, which for the LBH benchmark set is always positive for our approach and always negative for different version of the methods presented in ref. 6. Tentatively, this may be attributed to some missing (configurational) contributions in UM-VT and/or to our strict separation of harmonic vibrational terms and conformational terms. The latter mainly concerns low frequency modes that are correlated to conformational transitions and which were a key motivation for the mRRHO method with the rotor cut-off *τ* as an adjustable variable. In other schemes, for example the one introduced by Zheng and Truhlar,^22^ attempts have been made to tackle this problem by explicitly combining the rotational, vibrational, and conformational partition function.

#### Linear alkanes

**Fig. 5:**
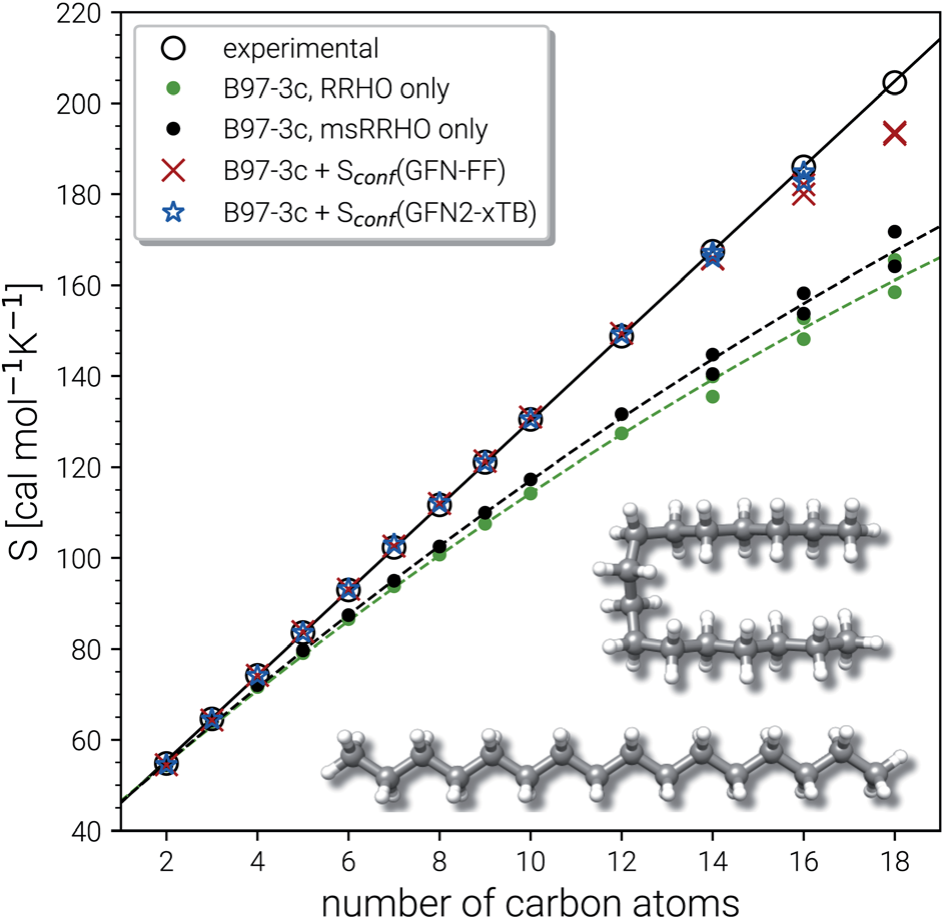
Parity plot for calculated and experimental entropies for *n*-alkanes from ethane to octadecane. All values correspond to B97-3c *S*_msRRHO_, either combined with GFN2-xTB or GFN-FF *S*_conf_, or without the conformational contribution. For C_14_H_30_ up to C_18_H_38_ two values are shown each, which correspond to the competing linear and folded global minima (see text for details). As example the folded and linear minimum energy conformers for hexadecane are depicted.

Computational and accuracy limits of the presented approach are explored for the example of *n*-alkanes of increasing size, up to C_18_H_38_ (see Fig. 5). Such extremely large flexible systems have not been considered before quantitatively.

The experimental entropy values^79,80^ show a strict linear increase with the number of carbon atoms and the reproduction of this relation represents a challenging task for theoretical methods. Both the RRHO as well as the msRRHO models increasingly underestimate the entropy with growing system size leading to a strongly non-linear behavior and errors of more than 20% for the largest alkanes considered. The major part of this difference can be accounted for by *S*_conf_. In fact, up to tetradecane (C_14_H_30_), the computed values are all still within chemical accuracy of 3 cal mol^−1^ K^−1^ upon adding the conformational term. However, other effects start to come into play at this system size. The global minimum of C_14_H_30_ and of smaller *n*-alkanes in the gas-phase always correspond to a linear (unfolded) structure. As intramolecular interactions, in particular London dispersion, become stronger with increasing system size, other conformers will be favored eventually. For C_14_H_30_ up to C_18_H_38_, a competing folded conformer (in which dispersion interactions are maximized) is observed.^82,83^ The folded conformers are energetically similar to the respective linear structure but differ strongly in their msRRHO entropy. Depending on the applied theoretical level, either conformation could be the global gas-phase minimum, which makes the choice of *S*_ref_ in eqn (11) ambiguous and could introduce errors. In the ideal case, the variations between different reference conformers in *S̄*_msRRHO_ and *S*_msRRHO_ would cancel and lead to the same conformational entropy regardless of the chosen global minimum. This is observed for C_18_H_38_ and *S*_conf_ calculated at the GFN-FF level and would always be the case if *S̄*_msRRHO_ (see eqn (11)) is calculated at the same level as *S*_msRRHO_. For C_16_H_34_ variations between the different theory levels are larger and only the GFN2 conformational entropy for the folded conformer as reference is still within chemical accuracy. Nevertheless, accurate entropies of extremely flexible large alkanes have been consistently obtained for the first time and this can be considered as a major achievement even though some issues for C_18_H_38_ remain. The detailed reasons for the deviations for the “worst cases” C_16_H_34_ and particularly C_18_H_38_ are not fully clear at this point but originate tentatively from the *S*_conf_ part.

Technical size limitations of our approach should also be noted. The computational cost increases strongly with molecule size at high flexibility and can make the conformational entropy calculation unfeasible for larger molecules. At the GFN2 level, the *S*_conf_ calculation for C_16_H_34_ already takes a few hundred hours of computation time, and hence, we did not attempt to calculate C_18_H_38_ at this level of theory. With the much cheaper GFN-FF method, on the other hand, the entropy for both C_16_H_34_ and C_18_H_38_ can still be computed roughly “over night” on a standard CPU node with 14 cores. Somewhat larger (up to 100–200 atoms) but less flexible molecules (*e.g.*, typical drugs, see Section 4.4) are also feasible at the GFN-FF level due to the shorter MD run times required. Neither of these system sizes can routinely be treated by DFT based MF approaches. In summary, the combination of *S*_msRRHO_ calculations with the specialized conformational sampling procedure for *S*_conf_, and the *S̄*_msRRHO_ averaging performs excellently and is on par with or even better than complicated and computationally demanding mode based approaches. Improvements of our approach may be necessary for molecules with a very large number of internal rotors at least if absolute values are considered and hence, a beneficial error compensation is not given.

### 4.3 Benchmarking heat capacity

**Fig. 6:**
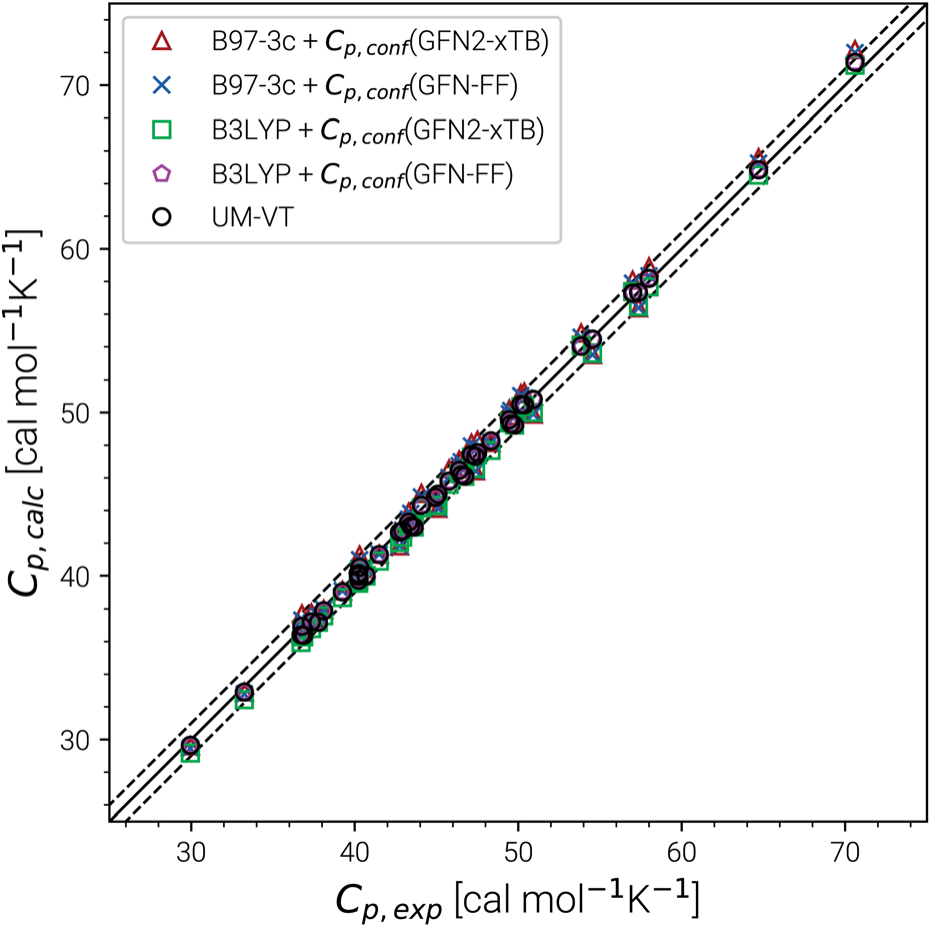
Parity plots for calculated and experimental heat capacities for a subset of the LBH set. Method combinations of B97-3c and B3LYP-D3/def2-TZVP *C*_p,msRRHO_ values with GFN2-xTB and GFN-FF *C*_p,conf_ values are shown. UM-VT values were taken from ref. 6.

Heat capacities and enthalpies (see eqn (13) and (14)) depend less strongly on the ensemble partition function than the entropy. Hence, it is sufficient to calculate *C*_p_ and enthalpies [*H*(*T*) − *H*(0)] only for a single converged ensemble without extrapolation. The performance of our approach was evaluated on a subset of the LBH benchmark with 44 experimental heat capacities for linear and branched alkanes at different temperatures between 300 and 500 K. For reference, we again compare with the UM-VT results provided in ref. 6. Parity plots for the comparison with experimental data are shown in Fig. 6 and the corresponding statistical data are given in Table 3.

**Table 3:** Mean deviation (MD), mean average deviation (MAD), root-mean-square deviation (RMSD) and standard deviation (SD) for heat capacities obtained at different theoretical levels in comparison to experimental data. All values are given in cal mol^−1^ K^−1^

*C*_p,RRHO_	B97-3c		B3LYP-D3/TZ		UM-VT^a^
*C*_p,conf_	GFN-FF	GFN2-xTB	GFN-FF	GFN2-xTB	
MD	0.05	0.17	−0.39	−0.11	−0.05
MAD	0.47	0.57	0.47	0.25	0.68
RMSD	0.58	0.69	0.54	0.32	0.78
SD	0.58	0.68	0.38	0.31	0.79

a Values taken from ref. 6.

Excellent performance is achieved for all assessed methods with RMSDs and SDs (much) smaller than 0.7 cal mol^−1^ K^−1^. In Fig. 6, virtually all data points are within an error range of 1 cal mol^−1^ K^−1^. The choice of the theoretical level used for the msRRHO calculations seems to be less important as both B97-3c and B3LYP-D3 perform well. Looking at the corresponding mean deviations B97-3c tends to slightly overestimate *C*_p_ while B3LYP-D3 shows the opposite trend. This is attributed to the choice of the frequency scaling factor and the cut-off value *τ*, which were adjusted for the computation of entropies. Accordingly, the results could be seen as further evidence for the conceptional validity of this treatment. At ambient temperature absolute values of heat capacities are smaller than absolute values for entropies. The corresponding conformational contributions are mostly not the accuracy bottleneck for the heat capacities but can be significant at lower temperatures. For example in the LBH subset, the largest *C*_p,conf_ values are obtained only for the most flexible systems (*n*-heptane, *n*-octane) and even then it accounts only to about 2–3 cal mol^−1^ K^−1^. However, it should be noted that the errors in the standard RRHO treatment will quickly exceed the desired 3 cal mol^−1^ K^−1^ range.

#### Temperature dependence of the heat capacity

**Fig. 7:**
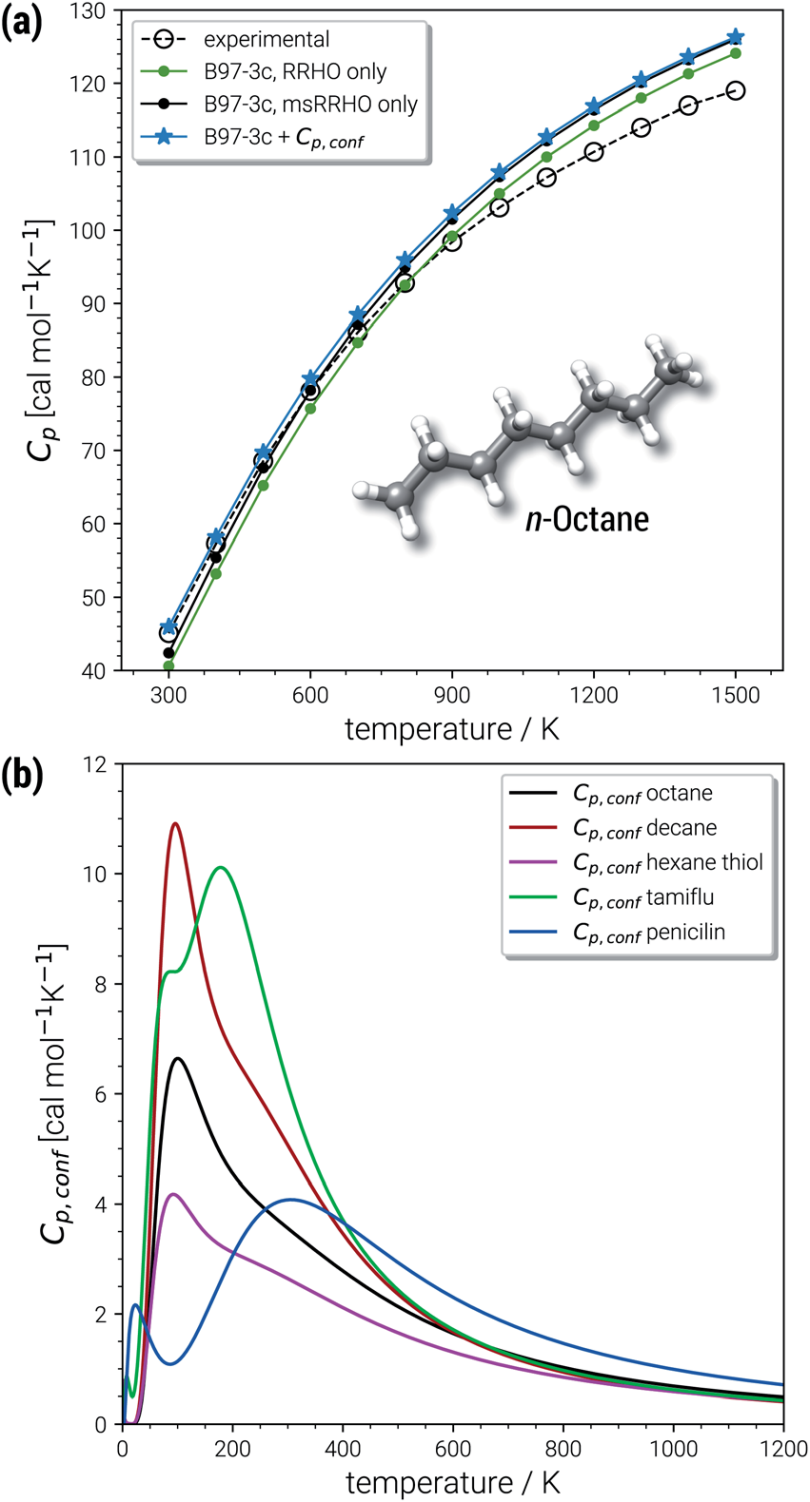
(a) Heat capacities calculated for *n*-octane in the temperature range 300 to 1500 K and (b) temperature dependence of the conformational heat capacity shown for octane and other example molecules from the AS23 and CD25 sets. (ms)RRHO values correspond to the B97-3c level and CE were obtained at the GFN2-xTB level.

As *C*_p,conf_ converges to zero with increasing temperature (all conformers are equally populated for *T* → ∞), the accuracy of the calculated heat capacity for large *T* depends mostly on the underlying frequency calculation. *n*-Octane is shown as an example in Fig. 7a, in comparison with experimentally derived^84^ heat capacities for in the temperature range from 300 to 1500 K. For temperatures below 500 K, the RRHO approach systematically underestimates the *C*_p_ values, which is improved by the msRRHO treatment. To reach chemical accuracy for this temperature regime, adding the conformational contribution is mandatory. With increasing temperature the unmodified RRHO value starts to overestimate the experimental *C*_p_. Because the msRRHO treatment always increases the heat capacity in comparison to the RRHO value, no improvement is obtained with our approach for very high temperatures. For *n*-octane at 1500 K this leads to an overestimation of 7 cal mol^−1^ K^−1^ in comparison to experiment. However, it should be noted that the high temperature reference values in Fig. 7 are derived indirectly from low temperature experimental data^84,85^ and hence these data points may have a larger uncertainties than the low temperature ones. In fact, other references can be found that differ from the here shown data and are slightly closer to the computed values.^86^

In the chemically important temperature regime of up to 500 K, where our approach is very accurate, a significant conformational contribution to the total *C*_p_ value is obtained (for a few examples see Fig. 7b). The temperature dependence of *C*_p,conf_(*T*) is very characteristic for each molecular structure and may contain maxima/minima in the curves. Extrema of *C*_p,conf_(*T*) can be associated with large changes of the individual conformer populations and may be interpreted as conformational phase transitions. For a more general review of interpretations of PES related heat capacity features see the work of Wales (ref. 25). The linear chain-like molecules in Fig. 7b (decane, octane and hexanethiol) only have a single maximum in the range 100–200 K. Around 200 K, many folded, higher energetic conformations start to be populated, while at lower temperatures only very linear structures are obtained. The global maximum of *C*_p,conf_ depends on the molecule specific energetic distribution of the conformers within a given energy window. For example, the CE of hexanethiol and octane consist of about the same number of conformers (150 and 152 structures respectively within 6 kcal mol^−1^), but differ with regard to their relative conformational energies. Molecular characteristics become even more pronounced for complicated molecules, *e.g.*, tamiflu and penicilin, where often multiple extrema are obtained for *C*_p,conf_(*T*) (see Fig. 7b).

### 4.4 Case studies

#### Drug molecules

After demonstrating the excellent performance of the presented approach to calculate absolute entropies in Section 4.2, we now turn our attention to biochemically more important systems. The CD25 set is introduced, containing 25 commercial drug molecules with 28 to 98 atoms. For these molecules no experimental entropy and *C*_p_ values are available to compare with. Nonetheless also a purely theoretical investigation of the CE and respective entropies may yield important insights. Note that a comprehensive evaluation of the entropy for such important molecules with a highly accurate method is missing in the chemical literature.

**Fig. 8:**
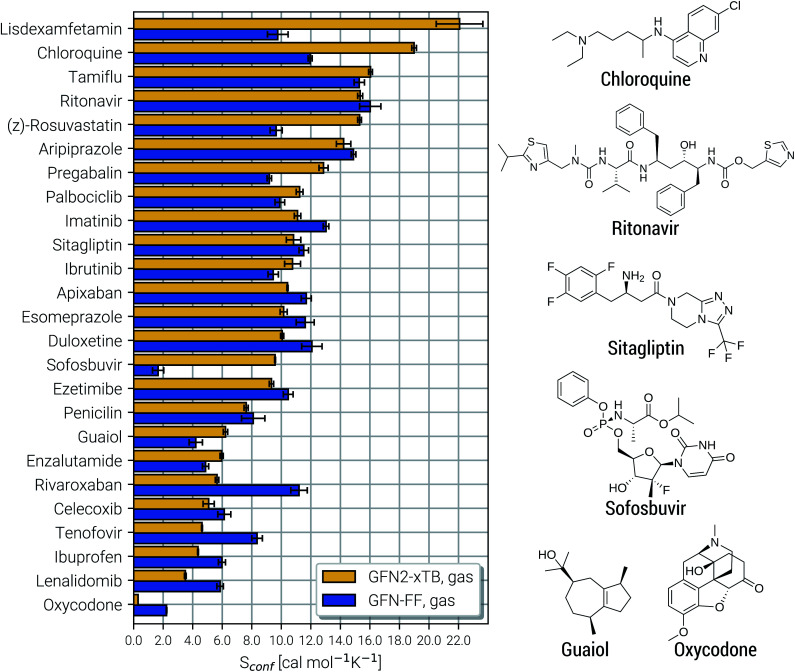
Calculated *S*_conf_ values for a set of 25 clinical drug molecules at the GFN2-xTB and GFN-FF levels of theory sorted according to increasing value. Averaged values (shown as horizontal bars) and their standard deviations (shown as errors) have been determined by multiple executions of the above described algorithm, as described in the text below. On the right side Lewis structures of some of the molecules are shown (see the ESI† for all molecules).

**Fig. 9:**
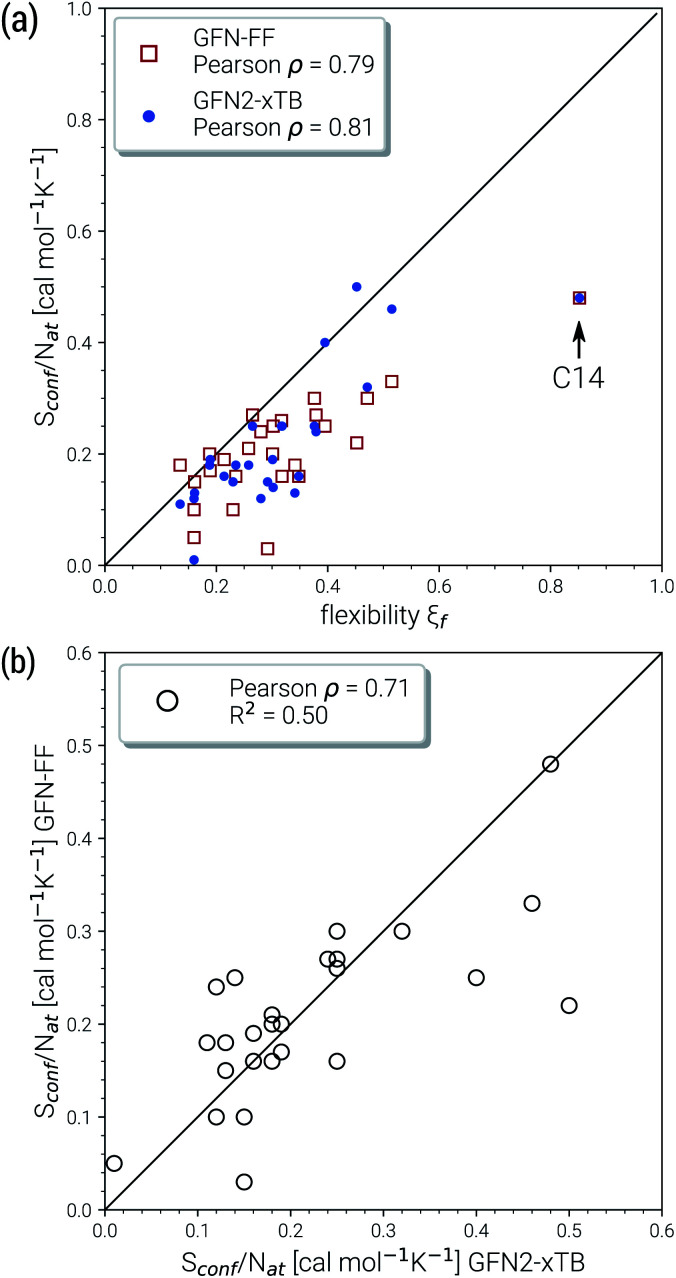
Correlation plots for the molecules of the CD25 set. The correlation between *S*_conf_/*N*_at_ and the empirical flexibility measure *ξ*_f_ is given in (a). Figure (b) shows the correlation of the *S*_conf_/*N*_at_ values at GFN-FF and GFN2-xTB level. The respective Pearson correlation coefficients *ρ* are shown in the legends.

Due to their similar size and elemental composition, similar *S*_conf_ values may be expected for typical drugs. This is not the case as can be seen from the entropies calculated for the CD25 set, shown for the GFN2-xTB and GFN-FF levels in Fig. 8. Conformational entropies in the CD25 set range from close-to-zero to over 20 cal mol^−1^ K^−1^. The reason for this is rooted in the very diverse and complicated PES of the molecules. Compared to the smaller and chemically rather similar molecules in the LBH and AS23 set, the molecules in the CD25 set show a variety of functional groups and intramolecular non-covalent binding motifs. This leads to a fine balance of covalent and non-covalent forces which characteristically shape the overall PES. Certain energy basins (a collection of related minima), for example, could be strongly favored because of intramolecular hydrogen bonding and thus reduce the overall number of energetically accessible minima. In such cases, an accurate description of the respective potentials is required and the computed *S*_conf_ value is strongly dependent on the underlying theoretical method. With a few notable exceptions, the conformational entropies calculated with GFN2-xTB and GFN-FF only differ by 1 to 2 cal mol^−1^ K^−1^ and therefore provide the same semi-quantitative description of the PES. The exceptions are cases in which GFN2 produces much larger CE (chloroquine, lisdexamfetamin, pregabalin, rosuvastatin, sofosbuvir) than GFN-FF, or *vice versa* (rivaroxaban, tenofovir). For the most rigid molecule (oxycodone), only a single conformer is significantly populated (*p*_*i*_ = 0.98 at 298 K) at the GFN2 level, while three conformers are populated at the GFN-FF level, resulting in a larger entropy. For the other cases with larger differences between both methods, the interpretation is difficult because of a large number of significantly populated structures (about hundreds) in the CE. A better understanding would be provided by an improved theoretical description, *i.e.*, the ensemble calculated by DFT or WFT but this is unfeasible due to the extremely high computational effort. Instead, one could refer to other qualitative descriptors when interpreting conformational entropies at a low theoretical level. Because the entropy is correlated with molecular structural features, one such descriptor could be the flexibility measure *ξ*_f_, which is used for determining the simulation length settings in CREST.^56^ This comparison of *ξ*_f_ and the *S*_conf_ is shown in Fig. 9 and in the ESI.† Note that conformational entropies must be normalized to system size (number of atoms *N*_at_) in order to be comparable in between molecules.

Both methods show a relatively high correlation with the empirical flexibility *ξ*_f_ in (Fig. 9a). The only outlier here is tetradecane, denoted as “C14” in the figure, which is chemically different from the drug molecules and was added only as an upper bound reference for the flexibility. When quantified *via* the well-known Pearson correlation coefficient *ρ*, it can be seen that GFN2-xTB (*ρ* = 0.81) corresponds slightly better with *ξ*_f_ than GFN-FF (*ρ* = 0.79). This indicates a better description of the few critical cases mentioned above at the tight-binding level. The correlation of *S*_conf_/*N*_at_ between the two methods (Fig. 9b, *ρ* = 0.71) again shows the intrinsic theory level dependence of the configurational entropy but is devoid from any deeper interpretation. Nonetheless, these examples demonstrate that the conformational entropy can be nicely correlated with purely structure based features of an ensemble or even empirical descriptors, which is why schemes such as the MIE^37^ and MIST^39^ have been proven to work comparatively well.

Finally, the CD25 set was employed to evaluate the robustness and reproducibility of the presented approach. As discussed above the stochastical nature of the MD runs leads to slightly varying results for different runs started on the same input structure. Hence, all of the 25 molecules were run several times in repetition and averaged to obtain *S*_conf_ and its standard deviation (SD) shown in Fig. 8. On average over the 25 systems, GFN2-xTB and GFN-FF yield SD values of 0.25 cal mol^−1^ K^−1^ and 0.35 cal mol^−1^ K^−1^ respectively. The only significantly larger SD of 1.6 cal mol^−1^ K^−1^ is obtained for the lisdexamfetamin molecule at GFN2-xTB level, which results from a large and complicated CE leading to convergence problems in 
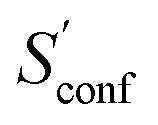
. In general GFN2-xTB has the more accurate PES of the two methods and produces more consistent results. Both GFN2-xTB and GFN-FF show reproducibility errors much below chemical accuracy and hence are appropriate for routine computations of *S*_conf_. The much shorter computation times of GFN-FF might favor its default application for large systems and also enables the averaging over multiple entropy calculations to eradicate statistical differences (which would be rather costly at the GFN2-xTB level).

#### Chemical applications

In this last section we give a few chemical examples, where absolute entropies are used to compute reaction entropies and Gibbs free energies.

**Fig. 10:**
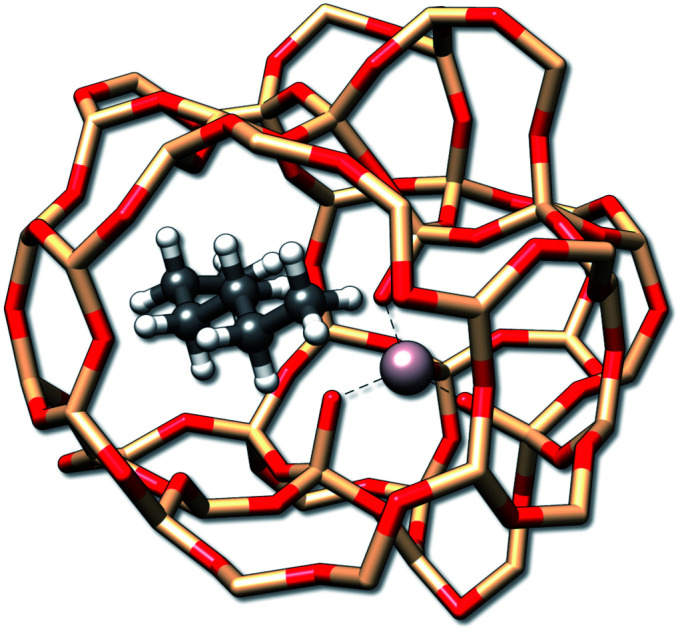
The *n*-hexane molecule adsorbed by a H-ZSM-5 zeolite. Hydrogen atoms used for the saturation of the zeolite have been omitted for better visibility.

Adsorption processes are important for a variety of applications, such as heterogeneous catalysis^87^ where the entropy change can be measured *via* calorimetric experiments. Here, a rather well studied class of reactions is the adsorption of *n*-alkanes onto zeolites.^88^ As an example the adsorption entropy of *n*-butane, *n*-pentane, and *n*-hexane (Fig. 10) in a H-ZSM-5 zeolite cut-out was calculated with GFN-FF.

For a given zeolite structure cut-out (*e.g.*, obtained from a crystal structure and saturated with hydrogen atoms) thermodynamic properties can be obtained with the (ms)RRHO approach. Sampling of the configurations in CREST then simply requires some additional geometrical constraints, as was discussed in previous work.^56,89^ This is necessary because the zeolite chunk shall mimic a solid and its structure would be strongly deformed or even broken by the metadynamic simulations and geometry optimizations at GFN level. The configurational problem is of course complicated by the combinatorial nature of different conformers at different adsorption sites, but in the present case the total system size is small enough to not pose major problems. Adsorption entropies are directly calculated from absolute entropies by Δ*S* = *S*_alkane/zeolite_ − *S*_alkane_ − *S*_zeolite_ (see Table 4) and assessed with respect to experimental values.

**Table 4:** Adsorption entropies (in cal mol^−1^ K^−1^) for small linear alkanes on H-ZSM-5 zeolite cut-outs, calculated fully at the GFN-FF level of theory. Experimental adsorption entropies were obtained from ref. 88

Adsorbed molecule	Δ*S*_msRRHO_	Δ*S*_conf_	Δ*S*_ads,calc._	Δ*S*_ads,exp._
*n*-Butane	−34.1	3.1	−31.0	−24.9
*n*-Pentane	−36.5	4.1	−32.4	−28.2
*n*-Hexane	−38.1	2.8	−35.3	−28.9

The final calculated Δ*S*_ads,calc._ shows deviations of only 4.2 to 6.4 cal mol^−1^ K^−1^ compared to experiment and show the same qualitative trend of adsorption strength (butane < pentane < hexane). While this trend is also reproduced already by *S*_msRRHO_, it is important to notice that the configurational contribution accounts for roughly 10% of the overall adsorption entropy and furthermore shifts Δ*S*_msRRHO_ in the direction of the experimental value. Because the zeolite is identical for all structures and configurations, all msRRHO entropies are similar and the term *S̄*_msRRHO_ consequently is ≪1 cal mol^−1^ K^−1^. Therefore the main part of Δ*S*_conf_ can be attributed to 
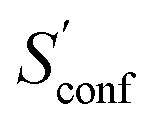
 and qualitatively interpreted. Here, *n*-butane has the smallest amount of conformers but many configurations (adsorption orientations) in the zeolite while it is *vice versa* for *n*-hexane, leading to a similar contribution of Δ*S*_conf_ ≈ 3 cal mol^−1^ K^−1^ in both cases. For *n*-pentane on the other hand, both the conformational and configurational space are large and hence it shows the largest Δ*S*_conf_ value of the three systems. The calculated Δ*S*_ads,calc._ are in very good agreement with experiment, considering that all results were obtained at a cost efficient force-field level and none of the values exceed a deviation of 2 kcal mol^−1^ at 298 K. Note that the full calculation for each of the final Δ*S*_ads_ values only took about 1.5–2 h on a standard desktop computer (4 cores on a Intel i7-7700K 4.2 GHz CPU).

A more common usage for *S*_conf_ is to improve the calculation of reaction free energies. The conformational entropies and enthalpies are converted to ensemble free energies *G*_conf_*via* the usual relation *G* = *H* − *TS* and can be added directly to the *G*_msRRHO_ values of all reactants and products of the reaction. In general, a significant change of the DOF in the course of the reaction can cause significant entropic effects and a non-negligible effect on the reaction free energy.

**Fig. 11:**
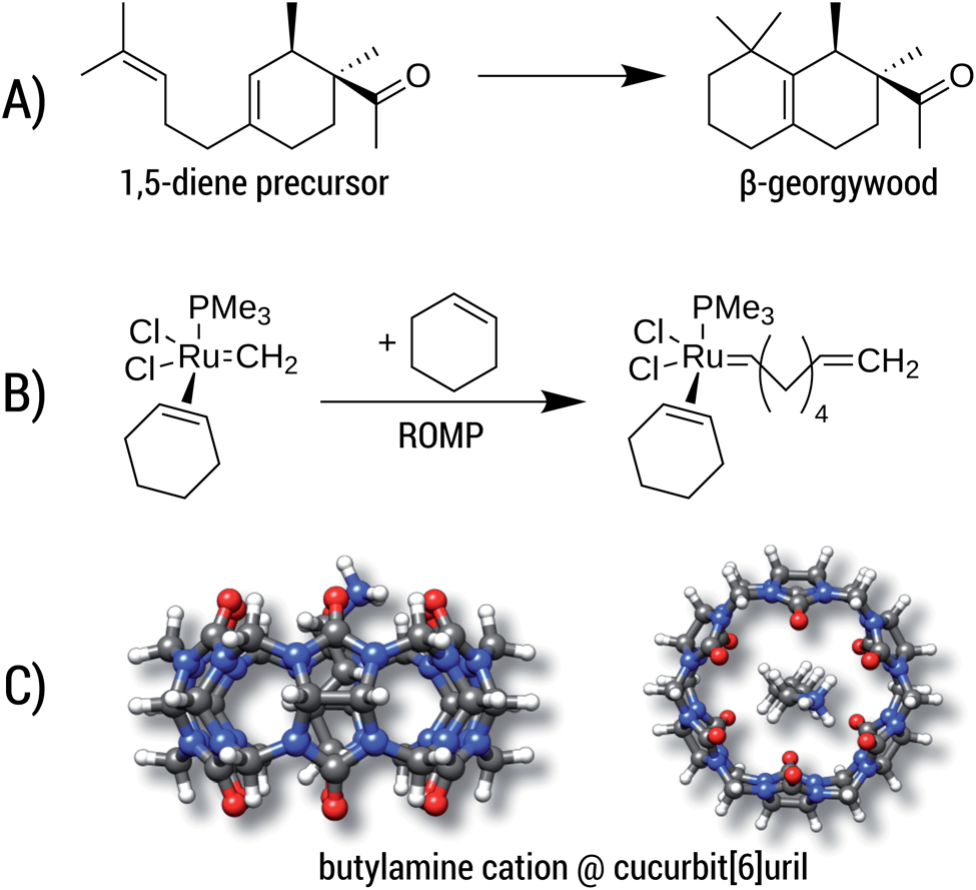
Example reactions with large entropic contributions. (A) Cyclization of a 1,5-diene to the β-georgywood compound, (B) simplified catalytic reaction of a ring-opening metathesis polymerization (ROMP), (C) complexation of butylammonium in cucurbit[6]uril.

Three examples (A, B, and C) are shown in Fig. 11 and the corresponding reaction energy differences are shown in Table 5.

**Table 5:** Energy differences for the reactions shown in Fig. 11. All values are given in kcal mol^−1^ and were obtained at the B97-3c level with conformational contributions calculated at GFN2-xTB level. Free energies correspond to 298.15 K

Reaction	Reaction energies		
	Δ*E*	Δ*G*	Δ*G* + Δ*G*_conf_
A	−15.0	−10.3	−8.7
B	−8.1	4.6	2.8
C	−82.0	−64.8	−64.3

Reaction A is the cyclization of a 1,5-diene into the perfume molecule β-georgywood.^90^ Ring-closure reactions are often associated with a decrease of DOF, and hence an entropic destabilization is expected. This view is supported by the computed free energies, where the addition of Δ*G*_conf_ decreases the reaction free energy from −10.3 kcal mol^−1^ to −8.7 kcal mol^−1^. For the typical “chemical accuracy” of 1 kcal mol^−1^, adding the conformational term would therefore be necessary. Note, that ring-closures are common in many syntheses and biochemical processes (*e.g.* terpene chemistry,^91^ or, as an example from a previous section, the synthesis of oxycodone^92^) and therefore will profit from a better description by our method.

Reaction B is a simplified catalytic reaction of a ring-opening metathesis polymerization (ROMP).^93^ ROMP was pioneered by the groups of Chauvin, Grubbs and Schrock and are among the most important catalytic reactions in industrial chemistry.^94,95^ The reaction free energy balance of B is positive as a result of the sterically undemanding PMe_3_ ligand, but nonetheless the influence of *G*_conf_ is nicely demonstrated. Here, due to a loss of DOFs (two reactants form one product molecule), Δ*G* becomes initially positive, which is counteracted by a DOF gain in *G*_conf_ of the product. The effect of the ensemble treatment has the same origin as in the ring-opening reaction A, but in this case favors the formation of the product by about 1.8 kcal mol^−1^. This example furthermore shows the capability of GFN2-xTB (and GFN-FF), which can be routinely be applied to transition-metal containing systems.

The influence of configurational entropy can also be studied for non-covalent associations. Reaction C shows the binding of butylammonium in cucurbit[6]uril.^96,97^ Binding affinities for small cations in cucurbiturils are well studied,^98^ but for more flexible guest molecules such as butylammonium, entropic effects may become important. The association free energy changes from −64.8 kcal mol^−1^ to −64.3 kcal mol^−1^ upon addition of Δ*G*_conf_ in the gas phase. On first sight, the increase of about 0.5 kcal mol^−1^ seems negligible compared to the large overall value of about −64 kcal mol^−1^. However, the latter value is quenched in solution^96,97^ to about −6.9 kcal mol^−1^ indicating that under more realistic conditions Δ*G*_conf_ is indeed relevant.

All the examples discussed in this subsection have been modelled in the gas-phase, but the extension to solutions is easily possible by using implicit solvation models. Inclusion of solvation effects will modify the PES and therefore produce different ensembles (and conformational entropies) than in the gas-phase. A direct impact of this would be noticeable, *e.g.*, for phase-partition coefficients like log *K*_ow_, which strongly depend on the respective ensemble.^99^ Technically, such calculations are straightforward and are investigated currently in our laboratory.

## 5 Conclusions

An automated workflow for the calculation of absolute molecular entropies is presented. The molecular entropy is a fundamental thermodynamic quantity necessary for a complete understanding of molecular interactions. The main component of the absolute entropy is usually obtained from vibrational frequency calculations in the RRHO approximation, which for medium sized molecules (50–100 atoms) often underestimates anharmonicities for low-frequency modes and is missing configurational contributions arising from many accessible low-energy conformations. In the presented approach both sources of error are treated by a separation of the molecular entropy into a configurational (conformational) part and the entropy arising from translational, rotational, and vibrational degrees of freedom. For the latter, vibrational frequencies were obtained at the B97-3c and B3LYP-D3/def2-TZVP DFT level, employing a modified and scaled RRHO approximation (termed msRRHO) with two adjustable parameters *τ* and *ν*_scal_. The conformational entropy is calculated from an ensemble of conformers using the well known Gibbs–Shannon entropy formula 
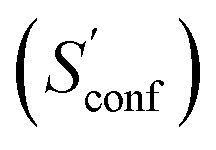
 and an population average over individual msRRHO contributions of the conformers (*S̄*_msRRHO_). We here make use of the fast and accurate GFN-FF and GFN2-xTB methods for the generation and energetic ranking of structures, driven by the recently introduced CREST program. The entire procedure is designed to work with only a few simple steps and minimal user input, which makes it routinely applicable to a broad range of systems.

The presented workflow was tested on a set of 62 experimental molecular gas phase entropies. An excellent performance (better than the chemical accuracy of 3 cal mol^−1^ K^−1^) was observed with MADs ranging from 0.73 to 0.92 cal mol^−1^ K^−1^ and SDs from 1.08 to 1.30 cal mol^−1^ K^−1^ respectively, depending on the combination of the DFT method with either GFN2-xTB or GFN-FF. Heat capacities were assessed on a set of linear and branches alkanes at different temperatures. The MAD and SD values are with 0.5 cal mol^−1^ K^−1^ even smaller than for absolute entropies but increase at very high temperatures >800 K. The presented method performs better than related yet computationally significantly more costly approaches and to our knowledge provides the smallest errors for molecular entropies ever reported in the literature. This includes large, extremely flexible *n*-alkanes up to octadecane for which an unprecedented accuracy for the absolute entropy in comparison to experiment of about 5% was obtained.

Biochemically important systems and chemical applications were discussed on the basis of set of 25 drug molecules and four reaction examples, including the calculation of adsorption entropies, two reaction free energies and a non-covalent association free energy calculation. For the drug molecules, a correlation of molecular flexibility and the entropy was observed. The examples revealed a significant contribution of the configurational terms to the overall free energy, often exceeding the magnitude of chemical accuracy. In the future, a more thorough study of these effects across a wide range of chemical reactions is desirable.

In general, GFN2-xTB was found to provide (as expected) a more consistent description of the PES and hence the conformational entropy than GFN-FF. However, as calculations of *S*_conf_ tend to get very expensive for larger systems at GFN2-xTB or higher theoretical levels, GFN-FF is strongly recommended as the standard approach in routine treatments on common desktop computers. In theory, the basic components of the proposed scheme are systematically improvable by a better description of the PES. The modular partition of the absolute value into ro-vibrational and configurational parts enables a convenient replacement of the different methods, which provides a starting point for future studies. This also includes the extension to implicit solvation models that will allow to investigate molecular entropy differences between the gas-phase and solution or between different solvents.

## Availability

The employed conformational search algorithm including the above described workflow for the calculation of molecular entropies was implemented in the recently published CREST program, version 2.11. The program (Linux/Unix compatible only) is available free of charge from GitHub (https://github.com/grimme-lab/crest). CREST requires access to the xtb binary, also available from GitHub (https://github.com/grimme-lab/xtb). Input geometries for the above calculations are available from https://github.com/grimme-lab/mol-entropy.

## Author contributions

Both authors contributed equally to the development of the theory, the software development, the conducted calculations and the writing of the manuscript.

## Conflicts of interest

There are no conflicts of interest to declare.

## Supplementary Material

SC-012-D1SC00621E-s001
