# Supplementary material for: Calculation of absolute molecular entropies and heat capacities made simple
Source: Chem Sci. 2021 Mar 25;12(19):6551–68. doi: 10.1039/d1sc00621e (PMC8139639; doi:10.1039/d1sc00621e)
Supplement: SC-012-D1SC00621E-s001 [file SC-012-D1SC00621E-s001.pdf]

Electronic Supplementary Material (ESI) for Chemical Science.
This journal is © The Royal Society of Chemistry 2021

Supporting Information: Calculation of absolute molecular entropies and heat capacities made simple

Philipp Pracht and Stefan Grimme*

Mulliken Center for Theoretical Chemistry, Institute for Physical and Theoretical Chemistry, University of Bonn, Beringstr. 4, 53115 Bonn, Germany

E-mail: grimme@thch.uni-bonn.de

Phone: +49-228/73-2351

1 Statistical error measures

Statistical measure for a set $x_1, \cdots, x_n$ of data points with references $r_1, \cdots, r_n$ are:

- Average:

$$\overline{x} = \frac{1}{n} \sum_{i}^{n} x_i \tag{1}$$

- Mean deviation (MD):

$$MD = \frac{1}{n} \sum_{i}^{n} (x_i - r_i) \tag{2}$$

- Mean absolute deviation (MAD):

$$MAD = \frac{1}{n} \sum_{i}^{n} |x_i - r_i| \tag{3}$$

- Standard deviation (SD):

$$SD = \sqrt{\frac{\sum_i^n |(x_i - r_i) - MD|^2}{n-1}} \tag{4}$$

- Root-mean-square deviation (RSMD):

$$RMSD = \sqrt{\frac{\sum_i^n |x_i - r_i|^2}{n}}, \tag{5}$$

- *Pearson* correlation coefficient (r_p):

$$r_p = \frac{\sum_{i=1}^{n} (x_i - \overline{x}_i)(r_i - \overline{r}_i)}{\sqrt{\sum_{i=1}^{n} (x_i - \overline{x}_i)^2 \sum_{i=1}^{n} (r_i - \overline{r}_i)^2}}, \tag{6}$$

2 Implementation, Algorithmic and Calculation Details

2.1 RMSD based metadynamics

The RMSD metadynamics (MTD) were introduced in Ref. 1 and are based on a bias potential

$$V_{bias} = \sum_i k_i \exp\left(-\alpha_i \Delta_i^2\right) \tag{7}$$

where Δ_i is the atomic RMSD[2] between a reference structure i and the calculated molecule. k_i and α_i empirical or automatically determined parameters that shape the potential. During a metadynamics simulation points on the simulated PES, i.e. snapshots of the MD simulation are saved for the calculation of Δ_i, which is then used to generate a repulsive V_{bias} contribution at the repective geometry. By a continuous collection and update of reference structures (from new snapshots) over the whole length of the simulation, V_{bias} will dynamically increase and form a history-dependent potential. This way previously found regions of the PES are blocked for the exploration and new conformers (PES minima) are found more safely.

As a new alternative we introduce another type of metadynamics, called static metadynamics (sMTD). In contrast to the MTD discussed in Refs. 1,3, this simulation is initialized with a given set of reference geometries and the MD will hence exhibit one global (and unchanged) V_{bias} potential. This version of MTD is more similar in nature to the well-known umbrella sampling or global optimization procedures. With regards to the PES sampling, sMTD has a less explorative character than MTD for finding the global minimum, but will more continously expand the conformational ensemble with new higherenergetic structures.

2.2 Molecular flexibility description

Many settings for the here discussed workflow are generated automatically and based on the individual structure of the investigated molecule. An important parameter is the molecular flexiblity, because it is directly related to the molecules accessible low-energy space. In Ref. 3 we proposed a molecular flexibility measure $\xi_{\mathrm{f,cov}}$, defined by

$$\xi_{\mathrm{f,cov}} = \sqrt{\frac{1}{N_{\mathrm{bonds}}}} \left(\sum_{i}^{N_{\mathrm{bonds}}} \left(1 - \mathrm{e}^{-5(\mathbf{B}_{\mathrm{AB}}-2)^{10}}\right)^2 \frac{4}{N_{\mathrm{A}}^{\mathrm{neigh}} N_{\mathrm{B}}^{\mathrm{neigh}}} \left(R_i^{(\mathrm{f})}\right)^2 \right)^{\frac{1}{2}} . \quad (8)$$

The summation over all *non*-terminal bonds i with the atoms A,B $\in i$ includes the Wiberg-Mayer bond order[4,5] (WBO) $\mathbf{B}_{\mathrm{AB}}$ between the two atoms. It is always obtained from a GFN0-xTB calculation because no WBO is accessible from FF data. $N_{\mathrm{A,B}}^{\mathrm{neigh}}$ are the numbers of neighboring atoms of A and B, respectively. $R^{(\mathrm{f})}$ is a predefined factor of value 1 if the bond i is not part of a ring and < 1 (depending on the ring size) otherwise. The measure $\xi_{\mathrm{f,cov}}$ works well for assigning a quantitative covalent (as indicated by the subscript addendum) flexibility, where values close to unity indicate an highly flexible system and values $\ll 1$ indicate rigid systems. It fails, however, for systems that are stabilized by non-covalent ineractions like hydrogen-bonds or dispersion. Reasonably sized organic moleculs, such as polypeptides, are often much more rigid as described by $\xi_{\mathrm{f,cov}}$, due to the formation of intramolecular hydrogen-bonding networks. Likewise, dispersion interactions are always present and might stabilize certain conformations, but do not contribute to the flexibility in Eq. 8. Therefore, a modified molecular flexibility is proposed that includes non-covalent contributions $\xi_{\mathrm{f,NCI}}$ from hydrogen bonds and dispersion to the total molecular flexibility

$$\xi_{\mathrm{f,tot}} = \frac{1}{2}\xi_{\mathrm{f,cov}} + \frac{1}{2}\left(\xi_{\mathrm{f,NCI}}\xi_{\mathrm{f,cov}}^{\frac{1}{2}}\right) . \quad (9)$$

Non-covalent interactions are quantified from the total hydrogen-bond energy E_{HB} and D4 dispersion energy E_{disp}, *relative* to the respective energies of a known reference system. In

order to be comparable to the reference, the energies must be normalized to the number of atoms N in the system. The final formulation for $\xi_{\mathrm{f,NCI}}$ then is given by

$$\xi_{\mathrm{f,NCI}} = 1.0 - \frac{1}{2}\left(\frac{E_{\mathrm{HB}}}{E_{\mathrm{HB,ref}}} + \frac{E_{\mathrm{disp}}}{E_{\mathrm{disp,ref}}}\right)\left(\frac{N}{N_{\mathrm{ref}}}\right)^{-1} \quad . \tag{10}$$

The energy contributions E_{HB} and E_{disp} are readily available from a simple GFN-FF singlepoint energy calculation. Respective reference contributions $E_{\mathrm{HB,ref}}$ and $E_{\mathrm{disp,ref}}$ are then assumed to be a calibration standard for all further computations of the molecular flexibility. As a reasonably flexible reference in which NCI interactions are impotant, the crambin protein was chosen for the calculation of $E_{\mathrm{disp,ref}}$ and $E_{\mathrm{HB,ref}}$.

2.3 Rotamer numbers

One of the key assumptions in the proposed scheme is that every contributing conformer i can be effectively represented by a number of energetically degenerate rotamer structures with its degeneracy number g_i. This number is composed of three parts

$$g_i = \frac{g_{rot}\, g_{core}}{g_{sym}} \quad , \tag{11}$$

where g_{rot} is a factor arising from single-bond rotations, g_{core} denotes a factor resulting from complex inversion and g_{sym} includes the molecular symmetry into g_i. Here, the factor g_{rot} is a constant that is the same for all *unique* conformers and (pseudo-)enantiomers. All conformers of a molecule have the same number of rotatable groups, each resulting in a fixed prefactor equal to the number of equivalent nuclei exchanged by the rotation (i.e., 3 for methyl, 2 for phenyl, 5 for η^5-$C_5H_5^-$, and so forth). We assume this factor to be constant for a given molecules since all combinations of the rotations would be observed at some point in time ($t \to \infty$). The factor g_{core} results from more complicated inversion-type processes that are responsible for the generation of other degenerate structures such as (pseudo-)enantiomers of a conformer. g_{core} is unique for every conformer since it is linked to the molecular symmetry

of the respective structure.

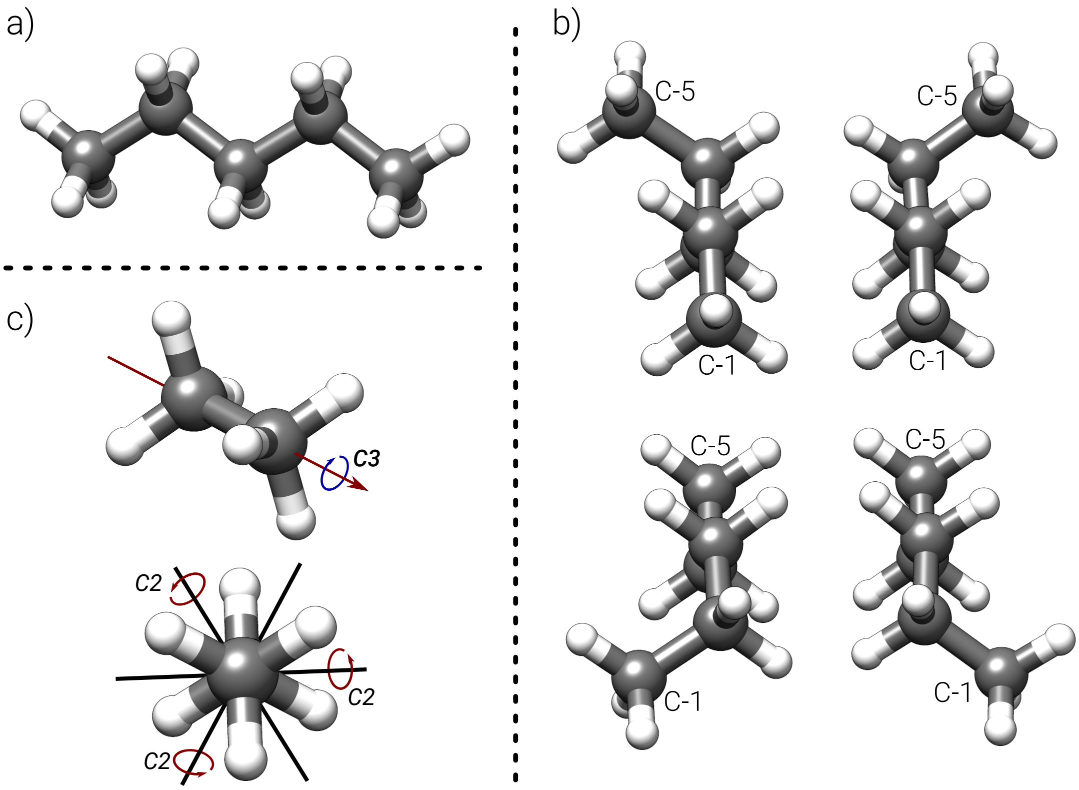

Figure 1: Structures of ethane and *n*-pentane. a) Lowest conformer of *n*-pentane in the gas-phase. b) Second lowest conformer of *n*-pentane. The conformer has four different pseudo-enantiomers, for better distinguishability the first and fifth carbon atom are labeled as such. c) Main symmetry elements within the ecliptic (D_{3d}) ethane molecule.

As an example the two lowest conformers of the n-pentane molecule are shown in Fig. 1a and 1b. With two terminal methyl groups n-pentane has a rotamer degeneration of $g_{rot} = 3^2$. The lowest conformer of n-pentane in the gas-phase has C_{2v} symmetry and no other enantiomeric structures exist ($g_{core} = 1$). In the second conformer, however, one of the terminal methyl groups is slightly twisted resulting in a total of four different (pseudo-)enantiomers ($g_{core} = 4$). Hence, 9 rotamers are to be expected for the lowest conformer of n-pentane, but there are 36 degenerate rotamers for the second conformer.

The rotamer number g_i also depends on the molecular symmetry. If symmetry operations exist that coincide with some of the rotations included in g_{rot} and can impose a nucleus on

itself, g_i has to be reduced by a factor g_{sym}. A simple example is the ethane molecule as shown in Fig. 1c. With two terminal methyl groups one could expect 3^2 rotamers, but since ethane has D_{3d} symmetry (D_{3h} for the eclipsed form) there are only three different rotamers for the molecule. Here, g_{rot} equals the symmetry number of the primary rotation axis. Other examples are neopentane (T_d), isobutane (C_{3v}), or ferrocene (D_{5d}/D_{5h}). For most molecules g_{rot} simply is unity and is only important for high symmetry cases.
The rotamer number g_i is generated automatically from the CRE obtained by a conformational search as implemented in the `CREST` program and information of chemically equivalent nuclei. Nuclear equivalencies are obtained as a by-product of the conformational search directly from the structure comparison as described in Ref. 6. For the identification of rotational groups, the topology of the molecule is set up for the lowest energy conformer and analyzed. Herein, the topology can be either based on quantum chemical data (covalent bond orders) or just set up directly from the coordination numbers (CNs). Molecular rings are identified in a graph representation of the topology, using an custom depth-first all-pair-shortest-path algorithm. Rotational groups are obtained form groups of equivalent nuclei and must obey some simple heuristic rules:

- The equivalent nuclei must be connected to a common neighboring atom.
- The neighboring atom may have a maximum of one neighbor other than the equivalent nuclei to be considered "freely rotatable". (An alternative definition via the WBO is possible).
- Rotations from different groups of equivalent atoms in the *same* ring must only be counted once to avoid double counting.
- The rotation number of the group is equivalent to the number of its members (i.e., the equivalent nuclei).

These rules work recursively (e.g., a *tert*-butyl group results in 3^4 rotamers), but special

consideration has to be paid to freely coordinated rings (e.g., Cp in ferrocene), which will not be discussed here any further.

The factor g_{core} is generated from a Cartesian RMSD comparison[2] of all structures in a CRE that belong to the same conformer. In this comparison all atoms that are rotationally equivalent must be neglected in the RMSD, leading to the identification of all the different "core" structures for each conformer, i.e., its (pseudo-)enantiomers. For an example again see Fig. 1b. The key assumption for this is, that the conformational search was able to generate all relevant enantiomeric structures of a single conformer at least once.

2.4 Single point hessian procedure

Within the described workflow and the calculation of the $\overline{S}_{msRRHO}$ population average, the entropy for a reference structure $S_{msRRHO,ref}$ has to be calculated. For consistency this reference term has to be calculated at the same level of theory as the population average in $\overline{S}_{msRRHO}$, that is GFN2-xTB or GFN-FF. A geometry optimization at this level might lead to an alteration of the frequencies and hence calculated entropy. On the other hand, if calculated directly for the DFT reference geometry, there is a high probability to observe imaginary modes because the DFT geometry will not necessarily be a minimum on the GFN2 or GFN-FF PES. To account for this problem in a "best of two worlds" approach we employ a new procedure called single point hessians (SPH). Details of the SPH approach will be published elsewhere,[7] but basically it works by applying an additive potential[1,3] similar to Eq. 7 above,

$$V_{SPH} = k \exp\left(-\alpha\Delta^2\right) \ , \tag{12}$$

where Δ is the atomic RMSD[2] between two molecular structures, and k and α define the potential shape. Within the SPH procedure k and α are calculated automatically in an iterative process, by repeatedly calculating the RMSD between the DFT input structure and a GFNn-xTB or GFN-FF reoptimized structure and updating V_{bias}, until no change in the geometry is observed. This essentially reshapes the PES at GFN2-xTB (or GFN-FF) level and removes any imaginary modes for frequencies calculated directly for the DFT geometry. Entropies calculated with frequncies from SPH resemble those at the DFT level, but retain a slight level of theory dependent shift, which makes them compatible with $\overline{S}_{msRRHO}$. With regards to computational cost the SPH approach is much cheaper than calculating frequencies at the DFT level, but more expensive as standard GFN2-xTB or GFN-FF Hessian calculations.

3 Test Sets

3.1 The LBH test set

Table 1: Absolute entropies for the LBH benchmark[8] set. Entropies are given for a combination of S_{msRRHO} entropy calculated at DFT (B97-3c or B3LYP-D3/def2-TZVP) and S_{conf} calculated at a lower (GFN2-xTB or GFN-FF) level. Mean deviation (MD), mean average deviation (MAD), root-mean-square deviation (RMSD) and standard deviation (SD) are given below. "plain" values correspond to msRRHO without S_{conf}. All values correspond to cal mol^{-1} K^{-1}.

			**B97-3c**			**B3LYP-D3/TZ**			UM-VT[a]
		experiment	plain	GFN-FF	GFN2-xTB	plain	GFN-FF	GFN2-xTB	
1	ethane	54.79	54.48	54.50	54.51	54.48	54.49	54.49	54.75
2	propane	64.61	64.32	64.36	64.52	64.28	64.28	64.44	64.57
3	n-butane	74.21	72.07	74.21	74.14	72.07	74.05	74.08	74.21
4	isobutane	70.63	70.22	70.25	70.35	70.20	70.21	70.25	70.22
5	n-pentane	83.55	79.71	83.76	83.53	79.74	83.72	83.83	83.56
6	isopentane	82.16	80.05	82.19	82.10	80.00	82.12	82.25	81.51
7	neopentane	73.14	72.86	72.87	73.02	73.01	72.97	73.03	73.18
8	n-hexane	92.94	87.39	93.12	93.18	87.52	93.30	93.39	93.49
9	2,2-dimethylbutane	85.66	85.91	85.95	86.07	85.70	85.81	85.64	84.97
10	2,3-dimethylbutane	87.46	85.70	88.08	87.19	85.32	87.66	87.04	85.17
11	2-methylpentane	91.06	87.68	91.48	91.57	87.72	91.27	91.01	90.56
12	3-methylpentane	91.54	87.86	91.00	90.84	87.88	90.71	90.25	90.54
13	n-heptane	102.32	94.97	102.61	102.99	95.19	102.79	102.60	102.88
14	2,2-dimethylpentane	93.86	93.06	94.37	94.73	93.13	94.45	94.39	92.71
15	2,3-dimethylpentane	99.11	94.44	98.63	97.68	94.20	98.11	97.10	94.72
16	2,4-dimethylpentane	94.89	91.86	95.39	95.46	92.13	95.48	95.54	92.8
17	3,3-dimethylpentane	95.20	90.55	102.11[b]	96.48	90.46	101.62[b]	96.21	93.66
18	3-ethylpentane	98.37	93.61	99.33	97.71	94.04	99.42	98.16	96.25
19	2-methylhexane	100.50	95.24	100.92	100.76	95.46	100.83	100.46	100.48
20	3-methylhexane	101.84	95.47	102.52	101.89	95.61	102.57	101.29	99.94
21	2,2,3-trimethylbutane	91.63	92.56	92.65	92.08	92.52	92.43	92.21	90.06
22	n-octane	111.70	102.52	112.00	112.18	102.87	112.07	112.04	113.51
23	1-butene	73.58	71.22	74.55	73.33	71.14	74.65	73.17	73.36
24	1,3-butadiene	66.63	66.34	67.57	66.88	66.22	67.49	66.78	66.05
25	ethyl methyl ether	73.91	72.79	75.28	75.26	72.78	74.49	75.14	73.07
26	ethanol	67.07	64.58	66.65	66.28	64.57	66.79	66.24	66.41
27	propionaldehyde	72.75	70.01	73.03	72.44	70.05	72.38	72.87	72.73
28	2-butanone	81.12	79.63	84.22	83.74	80.19	84.70	83.86	80.63
29	acetic acid	67.75	69.97	70.04	69.98	69.08	69.60	69.11	68.06
30	propylamine	77.78	72.80	76.85	76.33	72.91	76.90	76.40	76.63
31	1-nitropropane	83.80	81.68	84.03	85.50	81.79	84.13	86.00	84.68
32	1-fluoropropane	72.85	70.68	72.67	73.04	70.65	72.61	72.96	73.11
33	1-chloropropane	75.43	73.27	75.52	75.67	73.17	75.38	75.49	75.54
34	1-bromopropane	79.07	76.04	78.27	78.45	75.93	78.21	78.36	78.08
35	ethyl methyl sulfide	79.64	77.13	80.13	79.54	76.92	80.15	79.14	79.41
36	methyl disulfide	80.16	78.97	80.14	80.99	78.61	79.79	80.52	80.12
37	ethanethiol	70.79	68.21	70.49	70.52	68.22	70.54	70.16	71.01
38	ethylene glycol	72.61	69.89	73.60	74.54	69.84	73.55	74.51	74.56
39	acrylic acid	73.54	71.90	73.29	72.99	71.71	73.08	72.61	72.18
	MD	—	-2.62	0.32	0.23	-2.63	0.23	0.09	-0.52
	MAD	—	2.79	0.59	0.65	2.74	0.60	0.65	0.86
	RMSD	—	3.46	0.84	0.91	3.39	0.85	0.93	1.24
	SD	—	2.29	0.79	0.89	2.18	0.83	0.93	1.14

[a]Values taken from Ref. 8. [b]Outlier neglected from the statistics.

3.2 The AS23 test set

Table 2: Absolute entropies for the AS23 benchmark set. Entropies are given for a combination of S_{msRRHO} entropy calculated at DFT (B97-3c or B3LYP-D3/def2-TZVP) and S_{conf} calculated at a lower (GFN2-xTB or GFN-FF) level. Mean deviation (MD), mean average deviation (MAD), root-mean-square deviation (RMSD) and standard deviation (SD) are given for the combined LBH+AS23 below. "plain" values correspond to msRRHO without S_{conf}. All values correspond to $\mathrm{cal\,mol^{-1}\,K^{-1}}$.

			B97-3c			B3LYP-D3/TZ		
		experiment	plain	GFN-FF	GFN2-xTB	plain	GFN-FF	GFN2-xTB
40	cyclohexane	71.27	71.33	71.31	71.31	71.26	71.27	71.24
41	cycloheptane	81.82	80.21	80.48	80.24	80.18	81.15	80.15
42	cyclooctane	87.66	86.51	87.52	88.61	86.47	87.78	91.44
43	perfluorheptane	158.88	152.65	164.32[b]	159.90	152.51	165.84[b]	160.04
44	2,2,4,4-tetramethylpentane	103.13	101.30	102.10	102.76	101.06	102.07	102.41
45	2,2,3,4,4-pentamethylpentane	108.70	106.47	107.14	106.12	106.35	106.99	105.77
46	3,3-Diethyl-2-methylpentane	116.00	109.55	121.75[b]	115.81	109.32	121.87[b]	115.00
47	dipropylether	100.98	92.59	103.52	103.86	93.00	103.84	103.81
48	triethylamine	96.90	92.78	101.57	101.63	93.31	102.57	101.05
49	1-heptanol	114.83	102.72	113.61	113.83	102.99	114.33	113.74
50	Thiacycloheptane	86.50	83.62	87.95	87.43	83.54	87.66	87.65
51	nonane	121.06	109.97	121.30	121.06	110.40	121.46	121.44
52	decane	130.44	117.31	130.96	130.51	117.79	131.02	130.77
53	dodecane	148.78	131.60	149.42	149.21	132.09	149.81	149.11
54	butyl-propyl-sulfide	117.90	105.43	118.31	120.15	105.58	118.54	120.05
55	1-hexanol	105.50	94.92	104.11	104.29	95.13	104.61	104.22
56	1-pentanol	96.20	87.41	94.90	95.06	87.42	95.17	94.78
57	1-butanol	86.80	79.75	85.60	85.79	79.75	85.84	85.65
58	1-propanol	77.10	72.12	76.37	76.21	72.03	76.47	76.02
59	1-butanthiol	89.70	83.39	89.67	89.17	83.46	89.74	89.22
60	1-pentanthiol	99.30	90.98	99.00	98.45	91.17	99.18	98.53
61	1-hexanthiol	108.60	98.62	108.50	107.80	98.89	108.64	107.95
62	1-heptanthiol	117.90	106.18	118.00	117.09	106.61	118.21	116.96
	LBH+AS23 errors							
	MD	—	-4.36	0.21	0.15	-4.32	0.24	0.07
	MAD	—	4.48	0.73	0.83	4.40	0.73	0.92
	RMSD	—	5.90	1.09	1.19	5.77	1.16	1.29
	SD	—	4.00	1.08	1.19	3.85	1.15	1.30

[b] Outlier neglected from the statistics.

3.3 Linear alkanes

Table 3: Entropies calculated for linear alkanes up to octadecane. All values correspond to $\mathrm{cal\,mol^{-1}\,K^{-1}}$.

alkane	carbon atoms	experiment	S_{msRRHO} B97-3c	S_{abs} B97-3c + GFN-FF	S_{abs} B97-3c + GFN2-xTB
ethane	2	54.79	54.48	54.50	54.51
propane	3	64.61	64.32	64.36	64.52
n-butane	4	74.21	72.07	74.21	74.14
n-pentane	5	83.55	79.71	83.76	83.53
n-hexane	6	92.94	87.39	93.12	93.18
n-heptane	7	102.32	94.97	102.61	102.99
n-octane	8	111.70	102.52	112.00	112.18
nonane	9	121.06	109.97	121.30	121.06
decane	10	130.44	117.31	130.96	130.51
dodecane	12	148.78	131.60	149.42	149.21
tetradecane(linear)	14	167.40	144.73	165.85	166.01
tetradecane(folded)	14	167.40	140.44	165.68	167.10
hexadecane(linear)	16	186.02	158.25	180.02	182.79
hexadecane(folded)	16	186.02	153.76	182.08	184.85
octadecane(linear)	18	204.50	171.72	193.19	—
octadecane(folded)	18	204.50	164.09	193.66	—

3.4 LBH set heat capacities

Table 4: Heat capacities for a subset of the LBH benchmark set. C_p are given for a combination of $C_{p,msRRHO}$ calculated at DFT(B97-3c or B3LYP-D3/def2-TZVP) and $C_{p,conf}$ calculated at a lower (GFN2-xTB or GFN-FF) level. Mean deviation (MD), mean average deviation (MAD), root-mean-square deviation (RMSD) and standard deviation (SD) are given below. All values correspond to cal mol^{-1} K^{-1}.

			B97-3c		B3LYP-D3/TZ		
	T / K	experiment	GFN-FF	GFN2-xTB	GFN-FF	GFN2-xTB	UM-VT[a]
isopentane	317.2	29.95	29.47	29.61	29.17	29.65	29.97
	358.2	33.25	32.79	32.91	32.44	32.91	33.04
	402.3	36.72	36.33	36.45	35.95	36.41	36.29
	449.2	40.24	39.98	40.08	39.57	40.00	39.59
	487.1	42.93	42.79	42.88	42.36	42.76	42.11
n-hexane	333.9	37.35	37.16	37.75	36.80	37.20	36.86
	365.2	40.22	40.22	40.72	39.81	40.13	39.54
	398.9	43.30	43.47	43.89	43.03	43.28	42.42
	433.7	46.39	46.74	47.09	46.27	46.46	45.33
	468.9	49.46	49.92	50.21	49.42	49.56	48.17
2,2-dimethylbutane	341.6	38.10	37.97	37.97	37.58	37.89	39.24
	353.2	39.25	39.10	39.10	38.70	39.04	40.33
	376.1	41.50	41.32	41.32	40.90	41.30	42.45
	412.4	44.95	44.77	44.77	44.32	44.82	45.73
	449.4	48.33	48.16	48.16	47.68	48.27	48.92
2,3-dimethylbutane	341.6	37.78	37.57	37.57	37.18	37.18	38.91
	371.2	40.69	40.45	40.45	40.02	40.02	41.58
	402.3	43.63	43.43	43.43	42.98	42.98	44.32
	436	46.73	46.57	46.57	46.10	46.10	47.17
	471.2	49.77	49.72	49.72	49.23	49.23	50.00
2-methylpentane	325.1	36.77	37.31	37.69	36.71	36.94	36.51
	362.2	40.30	40.98	41.27	40.33	40.52	39.79
	402.3	44.08	44.83	45.04	44.16	44.32	43.26
	436.2	47.14	47.95	48.12	47.28	47.42	46.10
	471.2	50.16	51.03	51.15	50.35	50.49	48.90
3-methylpentane	332.1	36.88	36.98	36.77	36.29	36.38	37.54
	367.6	40.25	40.46	40.20	39.74	39.76	40.54
	402.4	43.43	43.82	43.52	43.08	43.05	43.46
	436.2	46.52	46.98	46.67	46.23	46.18	46.23
	471.2	49.55	50.12	49.81	49.37	49.30	48.99
n-heptane	357.1	45.77	46.02	46.54	45.61	45.80	46.18
	373.2	47.51	47.84	48.30	47.42	47.55	47.66
	400.4	50.37	50.89	51.25	50.44	50.47	50.18
	434.4	53.85	54.58	54.84	54.10	54.06	53.29
	466.1	57.00	57.89	58.08	57.38	57.29	56.12
2,2,3-trimethylbutane	328.8	42.74	41.83	41.83	42.04	42.68	44.11
	348.9	45.09	44.13	44.13	44.30	45.00	46.22
	369.2	47.39	46.43	46.43	46.57	47.32	48.33
	400.4	50.92	49.90	49.90	50.01	50.81	51.49
	434.3	54.54	53.55	53.55	53.63	54.47	54.79
	461.8	57.36	56.39	56.39	56.46	57.33	57.36
n-octane	405.7	58.00	58.34	58.89	57.67	58.19	57.51
	462.5	64.70	65.25	65.58	64.52	64.84	63.38
	522.7	70.60	71.99	72.17	71.22	71.40	69.24
	MD	—	0.05	0.17	-0.39	-0.11	-0.05
	MAD	—	0.47	0.57	0.47	0.25	0.68
	RMSD	—	0.58	0.69	0.54	0.32	0.78
	SD	—	0.58	0.68	0.38	0.31	0.79

[a] Values taken from Ref. 8.

Table 5: Heat capacities for n-octane in the range of 300 up to 1500K. All values correspond to $\mathrm{cal\,mol^{-1}\,K^{-1}}$.

T / K	experiment[a]	$C_{p,RRHO}$	$C_{p,msRRHO}$	$C_{p,msRRHO}$+$C_{p,conf}$
300	45.10	40.6	42.4	45.95
400	57.30	53.2	55.4	58.17
500	68.55	65.2	67.6	69.71
600	78.10	75.7	78.2	79.80
700	86.10	84.7	87.2	88.47
800	92.80	92.5	94.9	95.94
900	98.40	99.2	101.5	102.38
1000	103.10	105.0	107.3	107.94
1100	107.20	110.0	112.2	112.75
1200	110.70	114.3	116.4	116.91
1300	114.00	118.0	120.1	120.50
1400	117.00	121.3	123.2	123.61
1500	119.00	124.1	126.0	126.31

[a]Values taken from Refs. 9,10.

3.5 CD25 set: drug molecules

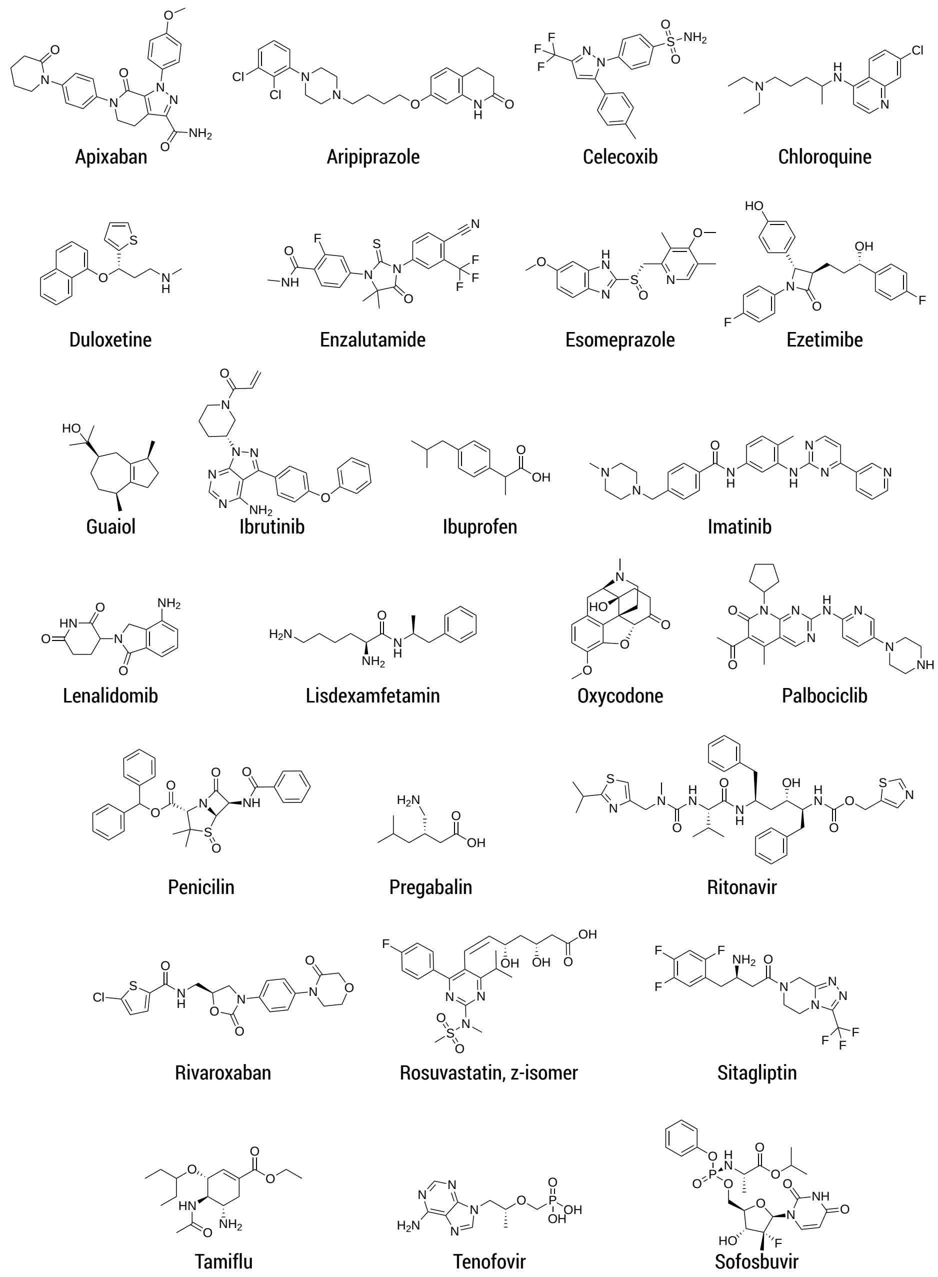

Figure 2: Lewis structures for all 25 molecules included in the CD25 set. Molecule names are given below the respective structure. Input structures are available from https://github.com/grimme-lab/mol-entropy.

Table 6: Comparison of a qualitative empirical flexibility measure ξ_f (see above) and the conformational entropy per atom for all molecules of the CD25 set at GFN2-xTB and GFN-FF level. Entropy values are given in $\mathrm{cal\,mol^{-1}\,K^{-1}}$ and are normalized to the number of atoms N_{at}. Values for tetra- and octadecane are given as further reference.

		S_{conf}/N_{at}	
molecule	ξ_f	GFN-FF	GFN2-xTB
Apixaban	0.188	0.20	0.18
Aripiprazole	0.317	0.26	0.25
Celecoxib	0.161	0.15	0.13
Chloroquine	0.395	0.25	0.40
Duloxetine	0.376	0.30	0.25
Enzalutamide	0.160	0.10	0.12
Esomeprazole	0.379	0.27	0.24
Ezetimibe	0.258	0.21	0.18
Guaiol	0.230	0.10	0.15
Ibrutinib	0.189	0.17	0.19
Ibuprofen	0.341	0.18	0.13
Imatinib	0.214	0.19	0.16
Lenalidomid	0.135	0.18	0.11
Lisdexamfetamin	0.452	0.22	0.50
Oxycodone	0.160	0.05	0.01
Palbociclib	0.235	0.16	0.18
Penicilin	0.301	0.20	0.19
Pregabalin	0.515	0.33	0.46
Ritonavir	0.348	0.16	0.16
Rivaroxaban	0.280	0.24	0.12
(z)-Rosuvastatin	0.318	0.16	0.25
Sitagliptin	0.265	0.27	0.25
Sofosbuvir	0.292	0.03	0.15
Tamiflu	0.471	0.30	0.32
Tenofovir	0.302	0.25	0.14
C14	0.852	0.48	0.48
C18	0.836	0.53	—

3.5.1 Empirical entropy estimates

As mentioned in the manuscript, the empirical formulation

$$S_{simple} = R \ln \left(N_{conf} \right) \tag{13}$$

is used in some studies[11,12] to estimate the conformational entropy. However, while this formulation may be used for very simple molecules, it breaks down for challenging energy surfaces. One could easily imagine a case where only a few conformers of an otherwise large ensemble contribute to the entropy (e.g., sofosbuvir at GFN-FF level), or an opposite case with many high-energetic conformers that individually contribute nothing, but in sum make a large part of the entropy (e.g., continuous ensembles, large n-alkanes). Population differences can thus lead to significant differences even for ensembles of same size, and would therefore not be captured by the approximation via N_{conf}. The approximation is further unable to capture vibrational entropy averages as in $\overline{S}_{msRRHO}$. Differences $\Delta S_{conf/simple}$ between this estimated and the fully converged entropy often exceed several cal mol^{-1} K^{-1} in either direction, which is shown for the CD25 in Tabs. 7,8 below.

Table 7: Conformational entropies and standard deviations (SD) calculated for the CD25 set from repeated CREST entropy sampling runs at GFN2-xTB level. Also shown is the simple entropy $S_{simple} = R\ln(N_{conf})$, estimated only from the number of conformers N_{conf} for each structure. All entropy values correspond to $\mathrm{cal\,mol^{-1}\,K^{-1}}$.

molecule	S_{conf}(GFN2-xTB)	SD	N_{conf}	S_{simple}	$\Delta S_{conf/simple}$
Apixaban	10.42	0.04	123	9.56	0.85
Aripiprazole	14.22	0.49	4273	16.61	-2.39
Celecoxib	5.08	0.38	10	4.51	0.57
Chloroquine	18.99	0.16	6499	17.45	1.54
Duloxetine	10.06	0.11	726	13.09	-3.02
Enzalutamide	5.96	0.11	23	6.20	-0.24
Esomeprazole	10.16	0.24	440	12.10	-1.93
Ezetimibe	9.33	0.15	664	12.91	-3.59
Guaiol	6.22	0.15	125	9.59	-3.38
Ibrutinib	10.76	0.54	470	12.23	-1.47
Ibuprofen	4.36	0.03	14	5.20	-0.84
Imatinib	11.10	0.22	851	13.41	-2.30
Lenalidomib	3.50	0.06	12	4.99	-1.49
Lisdexamfetamin	22.07	1.58	10044	18.31	3.76
Oxycodone	0.30	0.00	9	4.37	-4.07
Palbociclib	11.24	0.23	613	12.75	-1.52
Penicilin	7.62	0.14	193	10.46	-2.84
Pregabalin	12.86	0.31	870	13.45	-0.59
Ritonavir	15.34	0.17	11895	18.65	-3.31
Rivaroxaban	5.65	0.12	40	7.35	-1.69
(z)-Rosuvastatin	15.30	0.13	572	12.62	2.68
Sitagliptin	10.83	0.49	930	13.58	-2.75
Sofosbuvir	9.58	0.03	1756	14.85	-5.27
Tamiflu	16.04	0.13	8863	18.06	-2.03
Tenofovir	4.63	0.03	65	8.29	-3.66
average	—	0.25	—	—	-1.56

Table 8: Conformational entropies and standard deviations (SD) calculated for the CD25 set from repeated CREST entropy sampling runs at GFN-FF level. Also shown is the simple entropy $S_{simple} = R\ln(N_{conf})$, estimated only from the number of conformers N_{conf} for each structure. All entropy values correspond to $\mathrm{cal\,mol^{-1}\,K^{-1}}$.

molecule	S_{conf}(GFN-FF)	SD	N_{conf}	S_{simple}	$\Delta S_{conf/simple}$
Apixaban	11.69	0.34	262	11.07	0.62
Aripiprazole	14.89	0.16	3602	16.27	-1.39
Celecoxib	6.14	0.44	19	5.80	0.34
Chloroquine	11.95	0.14	4972	16.91	-4.97
Duloxetine	12.07	0.69	1157	14.02	-1.95
Enzalutamide	4.87	0.21	17	5.65	-0.78
Esomeprazole	11.62	0.61	490	12.31	-0.69
Ezetimibe	10.47	0.33	214	10.66	-0.20
Guaiol	4.21	0.45	67	8.35	-4.14
Ibrutinib	9.45	0.35	1110	13.94	-4.48
Ibuprofen	5.98	0.24	54	7.94	-1.96
Imatinib	13.03	0.19	1916	15.02	-1.99
Lenalidomib	5.86	0.22	9	4.37	1.50
Lisdexamfetamin	9.76	0.70	2429	15.49	-5.73
Oxycodone	2.23	0.02	28	6.61	-4.38
Palbociclib	9.90	0.33	571	12.61	-2.72
Penicilin	8.10	0.21	582	12.65	-4.55
Pregabalin	9.19	0.15	771	13.21	-4.02
Ritonavir	16.02	0.72	1467	14.49	1.53
Rivaroxaban	11.20	0.56	190	10.43	0.77
(z)-Rosuvastatin	9.65	0.41	628	12.80	-3.15
Sitagliptin	11.52	0.32	917	13.55	-2.03
Sofosbuvir	1.66	0.38	103	9.21	-7.55
Tamiflu	15.21	0.24	9559	18.21	-3.00
Tenofovir	8.36	0.36	236	10.85	-2.50
average	—	0.35	—	—	-2.30

References

(1) Grimme, S. Exploration of Chemical Compound, Conformer, and Reaction Space with Meta-Dynamics Simulations Based on Tight-Binding Quantum Chemical Calculations. *J. Chem. Theory Comput.* **2019**, *15*, 2847–2862.

(2) Coutsias, E. A.; Seok, C.; Dill, K. A. Using quaternions to calculate RMSD. *J. Comput. Chem.* **2004**, *25*, 1849–1857.

(3) Pracht, P.; Bohle, F.; Grimme, S. Automated exploration of the low-energy chemical space with fast quantum chemical methods. *Phys. Chem. Chem. Phys.* **2020**, *22*, 7169–7192.

(4) Wiberg, K. B. *Tetrahedron* **1968**, *24*, 1083–1096.

(5) Mayer, I. Bond order and valence indices: A personal account. *J. Comput. Chem.* **2007**, *28*, 204–221.

(6) Grimme, S.; Bannwarth, C.; Dohm, S.; Hansen, A.; Pisarek, J.; Pracht, P.; Seibert, J.; Neese, F. Fully Automated Quantum-Chemistry-Based Computation of Spin–Spin-Coupled Nuclear Magnetic Resonance Spectra. *Angew. Chem. Int. Ed.* **2017**, *56*, 14763–14769.

(7) Spicher, S.; Grimme, S. (2021), Single Point Hessian Calculations for Improved Vibrational Frequencies and Rigid-Rotor-Harmonic-Oscillator Thermodynamics, submitted.

(8) Li, Y.-P.; Bell, A. T.; Head-Gordon, M. Thermodynamics of Anharmonic Systems: Uncoupled Mode Approximations for Molecules. *J. Chem. Theory Comput.* **2016**, *12*, 2861–2870.

(9) Vansteenkiste, P.; Van Speybroeck, V.; Marin, G. B.; Waroquier, M. Ab Initio Calculation of Entropy and Heat Capacity of Gas-Phase n-Alkanes Using Internal Rotations. *J. Phys. Chem. A* **2003**, *107*, 3139–3145.

(10) Linstrom, E. P.; Mallard, W. NIST Chemistry WebBook, NIST Standard Reference Database Number 69. https://webbook.nist.gov/chemistry/, accessed December 18, 2020.

(11) Guthrie, J. P. Use of DFT Methods for the Calculation of the Entropy of Gas Phase Organic Molecules: An Examination of the Quality of Results from a Simple Approach. *J. Phys. Chem. A* **2001**, *105*, 8495–8499.

(12) Ghahremanpour, M. M.; van Maaren, P. J.; Ditz, J. C.; Lindh, R.; van der Spoel, D. Large-scale calculations of gas phase thermochemistry: Enthalpy of formation, standard entropy, and heat capacity. *J. Chem. Phys.* **2016**, *145*, 114305.
